# The Gut Microbiome and Colistin Resistance: A Hidden Driver of Antimicrobial Failure

**DOI:** 10.3390/ijms26188899

**Published:** 2025-09-12

**Authors:** Ionela-Larisa Miftode, Andrei Vâţă, Radu-Ştefan Miftode, Alexandru Florinel Oancea, Maria-Antoanela Pasăre, Tudoriţa Gabriela Parângă, Egidia Gabriela Miftode, Irina Luanda Mititiuc, Viorel Dragoş Radu

**Affiliations:** 1 Department of Internal Medicine II (Infectious Diseases), Faculty of Medicine, University of Medicine and Pharmacy “Grigore T. Popa”, 700115 Iasi, Romania; ionela-larisa.miftode@umfiasi.ro (I.-L.M.); andrei.vata@umfiasi.ro (A.V.); egidia.miftode@umfiasi.ro (E.G.M.); 2 “St Parascheva” Clinical Hospital of Infectious Diseases, 700116 Iasi, Romania; antoanela.pasare@gmail.com (M.-A.P.); tudorita.paranga@umfiasi.ro (T.G.P.); 3 Department of Internal Medicine I, Faculty of Medicine, University of Medicine and Pharmacy “Grigore T. Popa”, 700115 Iasi, Romania; alexandru.oancea@umfiasi.ro; 4 “St Spiridon” Emergency Hospital, 700115 Iasi, Romania; 5 Department of Urology, Faculty of Medicine, University of Medicine and Pharmacy “Gr. T. Popa”, 700115 Iasi, Romania; luanda.mititiuc@umfiasi.ro (I.L.M.); viorel.radu@umfiasi.ro (V.D.R.); 6 Department of Urology and Renal Transplantation, “C.I. Parhon” University Hospital, 700115 Iasi, Romania

**Keywords:** colistin resistance, *mcr* genes, gut microbiota, antimicrobial resistance (AMR), horizontal gene transfer (HGT), neonatal colonization, plasmid-mediated resistance

## Abstract

Colistin, a polymyxin antibiotic reintroduced as a last-resort therapy against multidrug-resistant Gram-negative bacteria, is increasingly being compromised by the emergence of plasmid-mediated colistin resistance genes (*mcr*-1 to *mcr*-10). The human gut microbiota serves as a major reservoir and transmission hub for these resistance determinants, even among individuals without prior colistin exposure. This review explores the mechanisms, dissemination, and clinical implications of *mcr*-mediated colistin resistance within the gut microbiota, highlighting its role in horizontal gene transfer, colonization, and environmental persistence. A comprehensive synthesis of the recent literature was conducted, focusing on epidemiological studies, molecular mechanisms, neonatal implications and decolonization strategies. The intestinal tract supports the enrichment and exchange of *mcr* genes among commensal and pathogenic bacteria, especially under antibiotic pressure. Colistin use in agriculture has amplified gut colonization with resistant strains in both animals and humans. Surveillance gaps remain, particularly in neonatal populations, where colonization may occur early and persist silently. Promising interventions, such as fecal microbiota transplantation and phage therapies, are under investigation but lack large-scale clinical validation. The gut microbiome plays a central role in the global spread of colistin resistance. Mitigating this threat requires integrated One Health responses, improved diagnostics for gut colonization, and investment in microbiome-based therapies. A proactive, multisectoral approach is essential to safeguard colistin efficacy and address the expanding threat of *mcr*-mediated resistance.

## 1. Introduction

The gut microbiota consists of a dense and dynamic ecosystem composed of trillions of microorganisms that perform essential physiological functions, including nutrient metabolism, immune modulation, and colonization resistance against pathogens [1]. However, beyond these beneficial roles, the gut also serves as a critical reservoir for antimicrobial resistance (AMR) genes—collectively known as the gut resistome [2,3]. Among these, resistance determinants against last-resort antibiotics such as colistin are of increasing global concern, given their implications for treating multidrug-resistant (MDR) bacterial infections [4,5].

Colistin (polymyxin E) is a cationic antimicrobial peptide that disrupts the outer membrane of Gram-negative bacteria by binding to lipopolysaccharides, particularly lipid A [6]. It is available in two main clinical forms: colistin sulfate, the active compound used mainly for oral and topical applications, and colistimethate sodium (CMS), an inactive prodrug administered intravenously or by inhalation that is converted in vivo to active colistin. CMS is the preferred formulation for treating severe systemic infections caused by multidrug-resistant Gram-negative pathogens such as carbapenem-resistant *Enterobacterales*, *Pseudomonas aeruginosa*, and *Acinetobacter baumannii*, while inhaled CMS is also used in cystic fibrosis [7]. Historically, its use was limited due to nephrotoxicity and neurotoxicity, but it has re-emerged as a vital therapeutic option for carbapenem-resistant *Enterobacterales* (CRE) and other MDR pathogens [8]. However, the clinical efficacy of colistin is now threatened by the emergence of mobilized colistin resistance (*mcr*) genes. First reported in China in 2015, the *mcr*-1 gene demonstrated that colistin resistance could spread horizontally via plasmids rather than chromosomal mutation alone, marking a paradigm shift in AMR surveillance and control [9]. Subsequent reviews have highlighted the rapid diversification of *mcr* variants across plasmid families and bacterial hosts, reinforcing their central role in the global dissemination of colistin resistance [5].

To date, at least ten *mcr* variants (*mcr*-1 to *mcr*-10) have been identified across diverse Gram-negative bacteria, particularly in *Escherichia coli*, *Klebsiella pneumoniae*, *Enterobacter* spp., and *Salmonella* spp. [10,11]. More recently, database annotations (e.g., NCBI Pathogen Detection) have flagged a putative *mcr*-11 variant (*mcr*-11.1), though this remains uncharacterized and lacks peer-reviewed confirmation. Thus, while ten validated *mcr* variants (*mcr*-1 to *mcr*-10) are firmly established, the existence of additional functional variants may be anticipated as genomic surveillance expands [12]. These genes encode phosphoethanolamine transferases that modify the bacterial lipid A structure, thereby reducing colistin binding affinity [13]. Their genetic and phenotypic diversity—including discovery timelines, plasmid contexts, and effects on colistin MICs—is summarized in Table 1, which illustrates both the widespread dissemination of *mcr*-1 and the more sporadic occurrence of later variants. Often embedded in conjugative plasmids—such as IncI2, IncX4, and IncHI2—*mcr* genes can readily disseminate among phylogenetically distant species within the gut microbiome [14,15]. Notably, their presence has been documented in individuals without any known exposure to colistin, including healthy children and adults [14,16].

Given the substantial heterogeneity in reported *mcr* prevalence across continents and sampling sources [11], a structured synthesis framework may be more informative than a single pooled estimate. First, studies should be stratified by setting (human, animal, environmental), methodological approach (culture-based versus metagenomic), sampling period, and population frame, with random-effects meta-analysis used to derive region-specific prevalence estimates accompanied by prediction intervals. Second, the development of a pragmatic risk stratification index for high-burden regions could integrate determinants such as colistin consumption, livestock density, sanitation and wastewater infrastructure, healthcare AMR burden, and travel connectivity. Third, conceptualizing the gut and its ecological interfaces as a meta-reservoir would enable the application of network analysis to metagenomic datasets, facilitating the identification of hub taxa and mobile genetic element backbones that disproportionately mediate *mcr* dissemination [32]. Together, these elements could provide an evidence-based foundation for prioritizing surveillance and guiding targeted interventions.

The gastrointestinal tract offers an ideal environment for the persistence and spread of resistance determinants. Commensal bacteria such as *E. coli*, *Citrobacter*, and *Enterobacter* spp. frequently harbor *mcr* genes without causing disease, effectively serving as asymptomatic carriers [2,33]. However, these genes can be horizontally transferred to virulent or opportunistic strains like *K. pneumoniae* during co-colonization events, especially under selective pressure from antibiotic use [34]. Such in-gut transmission events are well documented in vitro and in vivo, including among pediatric and neonatal populations [35].

Beyond clinical settings, colistin has a long history of extensive use in agriculture and veterinary medicine, dating back to the 1960s when it was introduced as a therapeutic agent and later adopted as a growth promoter in pig and poultry farming [36]. This large-scale, often sub-therapeutic, use created strong selective pressure that fueled the emergence and dissemination of plasmid-mediated *mcr* genes [37]. Resistant bacteria and plasmids originating from animal intestines can subsequently enter human microbiota through contaminated food, water, and direct contact, thereby completing a zoonotic and foodborne AMR cycle [38,39]. Environmental surveys have further detected *mcr*-positive *Enterobacterales* in livestock, slaughterhouse waste, aquaculture, retail meat, and wastewater treatment plants, highlighting the breadth of this dissemination [40,41]. Although colistin was never approved for agricultural use in some regions such as the United States, its widespread employment in Asia, Europe, and South America, coupled with international food trade, has contributed to the worldwide distribution of colistin resistance determinants [42].

Recent work has highlighted the ecological risks of combined agricultural and pharmaceutical pollutants in shaping resistance dynamics. A 2025 study demonstrated that co-exposure to the fungicide cyazofamid and colistin in a soil–animal–plant system not only exerted synergistic toxicity on earthworms and tomato plants but also disrupted microbial communities more severely than either agent alone. Importantly, such co-contamination enriched insertion sequences, plasmid-associated elements, and multiple antibiotic resistance genes (ARGs), suggesting that fungicide–antibiotic interactions may accelerate the mobilization and persistence of *mcr* and other resistance determinants in agroecosystems [43]. These findings align with recent metagenomic observations of rising *mcr*-9 prevalence, suggesting that environmental co-selectors may amplify the silent spread of low-level resistance determinants. Incorporating fungicide–antibiotic interactions into prevalence models and mitigation strategies will therefore be essential to refine risk estimates and address the evolving One Health challenge [43].

Given these multifaceted threats, surveillance data increasingly recognize the gut microbiome as both a reservoir and a transmission hub for colistin resistance. This has prompted urgent calls for integrated One Health responses that encompass human, animal, and environmental health sectors. Without such coordinated intervention, the spread of *mcr* genes could render colistin—and potentially other last-line antibiotics—ineffective in the near future [44]. Therefore, this study aims to provide a comprehensive overview of colistin resistance within the human gut microbiota, with a focus on the emergence and dissemination of plasmid-mediated *mcr* genes, their clinical and ecological implications, and the urgent need for integrated One Health strategies and microbiome-based interventions—particularly in vulnerable populations such as neonates.

This review was conducted as a narrative synthesis. To ensure transparency and minimize bias, we performed a structured literature search across PubMed, Scopus, and Web of Science for the period January 2015 to August 2025, using combinations of keywords such as “*mcr*”, “colistin resistance”, “antimicrobial resistance”, “gut microbiota”, “horizontal gene transfer”, and “decolonization”. Additional references were identified by screening the bibliographies of relevant articles. Only peer-reviewed, English-language publications were included, with exclusion of preprints and non-peer-reviewed material unless explicitly cited as emerging evidence. Articles were considered if they addressed the occurrence, mechanisms, transmission, surveillance, or mitigation of *mcr* genes in human, animal, or environmental contexts. While no formal PRISMA framework was applied due to the narrative scope, this structured approach aimed to balance breadth of coverage with reduction in selection bias.

### 1.1. Gut Microbiota as a Reservoir for Colistin Resistance Genes

Notably, commensal gut bacteria—especially *E. coli*, but also *Enterobacter*, *Citrobacter*, and others—frequently harbor *mcr* genes without causing disease, effectively acting as a reservoir for resistance [16,45]. Global surveillance studies have detected *mcr* genes in the gut microbiome of individuals on every inhabited continent [11,46]. For instance, a 2021 metagenomic analysis identified over 2000 putative *mcr*-like genes in gut bacterial genomes from 41 countries, highlighting their cosmopolitan distribution [46]. However, this study relied exclusively on sequence-based homology searches without functional validation, meaning that many of the predicted genes may not confer phenotypic resistance in vivo [46].

Importantly, *mcr*-positive plasmids are often found in asymptomatic hosts and can co-exist with other resistance genes, such as those encoding extended-spectrum β-lactamases (ESBLs), raising concern over gut flora as a source of future multidrug-resistant infections [46]. Healthy carriers have been documented, for example, nearly 10% of children in a Chinese cohort were found to carry *mcr*-1-positive *E. coli* [14]. Similarly, *mcr* genes have been identified in gut bacteria from healthy livestock and wildlife, supporting the One Health concept linking human, animal, and environmental reservoirs [11,46]. Alarmingly, data from Nigeria revealed *mcr* gene carriage in approximately 1% of fecal samples from mothers and newborns, with *Enterobacter* and *E. coli* as the most common carriers—despite minimal use of colistin in the clinical setting [35].

This diversity of hosts and bacterial species demonstrates how the gut microbiota provides a breeding ground for colistin resistance genes to persist and spread. Emerging evidence suggests that harboring *mcr* genes may even enhance bacterial fitness in the gut. For example, an in vivo murine study using isogenic *E. coli* variants with or without *mcr*-1 demonstrated that an extra-intestinal pathogenic *E. coli* carrying *mcr*-1 had a competitive colonization advantage, even in the absence of antibiotic selection [47]. The gene reduced host inflammatory responses and conferred cross-resistance to antimicrobial peptides, supporting long-term bacterial persistence and a more commensal-like lifestyle [33,47]. These findings suggest that *mcr* genes may be stably maintained in gut bacteria, not only due to antibiotic pressure but also because of fitness benefits, further entrenching the gut microbiota as a reservoir for colistin resistance.

In a recent metagenomic study, Avellán-Llaguno et al. profiled the gut microbiomes of urban cats (household, free-roaming, and free-range domestic groups), uncovering 890 ARGs—with the freest cats exhibiting the highest ARG and virulence factor diversity—thereby highlighting the role of companion animals in environmental and zoonotic resistome dynamics [48]. Moreover, wastewater surveillance in Georgia, USA, revealed *mcr*-9-positive *Serratia nevei* in sewage and chromosomal *mcr*-3-positive *Aeromonas jandaei* in effluent and surface waters [49]. *mcr*-9 was carried on conjugative IncHI2 plasmids with the qseB/qseC system, transferable to *E. coli*, and persisted in 12-day biofilms. Isolates showed high colistin MICs and multidrug resistance with additional ARGs. These findings highlight wastewater treatment plants as effective One Health sentinels and emphasize the need to monitor overlooked bacterial hosts for plasmid-mediated colistin resistance [49].

Multiple studies also highlight the importance of viewing the gut microbiota as a dynamic “meta-reservoir” where resistome stability is shaped by ecological interactions, co-resistance linkages, and horizontal gene transfer networks. For instance, network analyses of metagenomic datasets have identified key bacterial taxa acting as hubs for mobilizing resistance genes across species boundaries, suggesting that dissemination is not random but structured by microbial community architecture [50,51]. Systems biology approaches are now being applied to simulate the dynamics of resistance acquisition and loss under varying selective pressures, such as diet, antimicrobial exposure, and colonization density [52,53]. Such predictive frameworks may enable the quantification of resistance enrichment and help identify intervention points to disrupt the maintenance of *mcr* genes in the gut ecosystem.

### 1.2. Impact of Colistin on Gut Microbial Diversity and Composition

**Figure 1:**
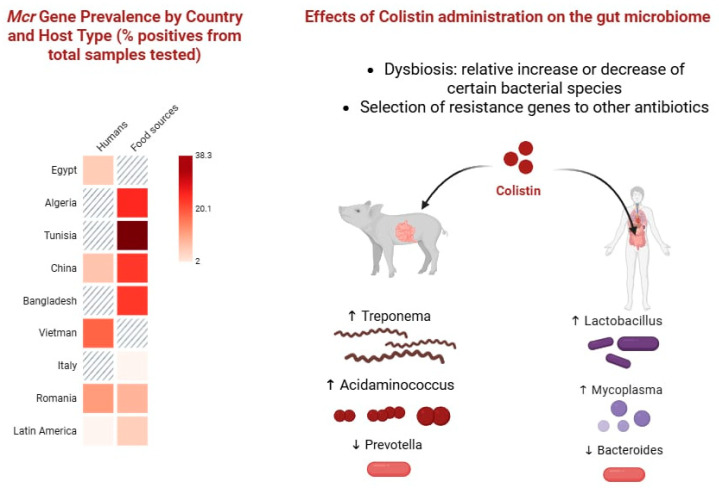
*mcr* gene prevalence and impact of colistin on the gut microbiome. (**Left**) Heatmap showing the prevalence of *mcr* genes in humans and food sources across different countries, expressed as the percentage of positive samples among those tested. (**Right**) Schematic overview of colistin administration and its effects on gut microbiota composition, including dysbiosis characterized by relative increases (↑) or decreases (↓) in specific bacterial taxa. Colistin exposure is associated with enrichment of *Treponema*, *Acidaminococcus*, *Lactobacillus*, and *Mycoplasma*, along with reductions in *Prevotella* and *Bacteroides*. Colistin use can also drive the selection of resistance genes to other antibiotic classes. Created in BioRender. Pasare, A. (2025) https://BioRender.com/vq0ic4j; Accessed on 31 August 2025.

Administration of colistin can significantly alter gut microbiome composition in both animals and humans. As a potent antibiotic against Gram-negative bacteria, colistin perturbs the balance of intestinal microbes, often killing susceptible strains and allowing intrinsically resistant or less affected populations to expand [54,55]. In food-producing animals, where colistin was historically used (e.g., as a growth promoter in piglets), studies document clear shifts in microbial diversity. For instance, a metagenomic analysis in pigs showed that colistin treatment changed the relative abundance of major gut bacteria: beneficial fermenters like *Prevotella* species sharply declined, while other genera such as *Treponema* and *Acidaminococcus* became more abundant [56] (Figure 1).

These changes corresponded with a measured decrease in overall microbial diversity in colistin-treated pigs compared to controls, suggesting that colistin may knock down dominant flora and enable a more even distribution of surviving species [56]. Colistin exposure also enriched the gut resistome in that study—expression of several antibiotic resistance genes (for tetracyclines, aminoglycosides, etc.) was upregulated after treatment, indicating that colistin can co-select for other resistance genes in the microbiota [56]. Similarly, in chickens, high rates of colistin use have been associated with a high prevalence of *mcr* genes in gut bacteria (e.g., a Bangladesh survey found *mcr*-1 in 25% of tested chicken fecal samples) [39], reflecting how routine colistin administration drives the expansion of colistin-resistant populations in animal intestines.

In humans, colistin is typically used systemically for severe infections rather than orally, but it can still impact the gut flora. Patients receiving intravenous or inhaled colistin may experience collateral effects on their gastrointestinal microbiome due to biliary excretion or swallowing of nebulized drug [57]. A laboratory simulation of the human intestinal microbiota exposed to colistin (alone and in combination with amoxicillin) demonstrated that colistin significantly alters microbial community structure [58]. Certain commensals (e.g., *Bacteroidetes* and some *Proteobacteria*) declined, while others (including *Lactobacillus* and *Mycoplasma* species) proliferated, reflecting a shift in genus-level composition [58,59]. The α- and β-diversity of the gut microbiome were measurably altered by colistin exposure in this model [59]. Moreover, the combination of colistin with another antibiotic was shown to induce a broader antibiotic resistome in gut bacteria, elevating the abundance of various resistance genes, and these microbiota changes were not easily reversible [58]. Clinically, patients receiving colistin therapy, especially in intensive care settings where broad-spectrum antibiotics are concurrently used, have developed intestinal carriage of colistin-resistant *Enterobacterales* [34].

Beyond these specific case reports, broader evidence supports the conclusion that colistin exposure causes gut dysbiosis—a disruption in microbial balance—which may be transient in many cases but can also persist longer-term depending on host and microbiome context, potentially leading to overgrowth of opportunistic pathogens and enhanced horizontal gene transfer [60]. Animal and human studies alike have demonstrated that colistin reduces microbial richness, disrupts functional pathways such as short-chain fatty acid production, and facilitates the emergence or persistence of resistant organisms [27,55,61,62]. These findings bring attention to a key point: colistin exerts a strong ecological pressure on the gut microbiome, and its use—whether in agriculture or clinical care—can unintentionally cultivate an intestinal environment that favors colistin-resistant microbes.

Available evidence indicates that colistin-induced perturbations of the gut microbiota may be partially reversible after treatment cessation, but the extent and pace of recovery depend on host factors (age, baseline microbiota diversity, immune status), treatment characteristics (dose, duration, co-administration of other antimicrobials), and environmental exposures such as diet or colonization pressure. Moreover, Guo et al. demonstrated that colistin modulates pig gut microbiome composition, suggesting that exposures shift communities—but also highlighting the dynamic nature of those changes, which can revert over time [56].

### 1.3. Horizontal Gene Transfer of Colistin Resistance Genes in the Gut

**Figure 2:**
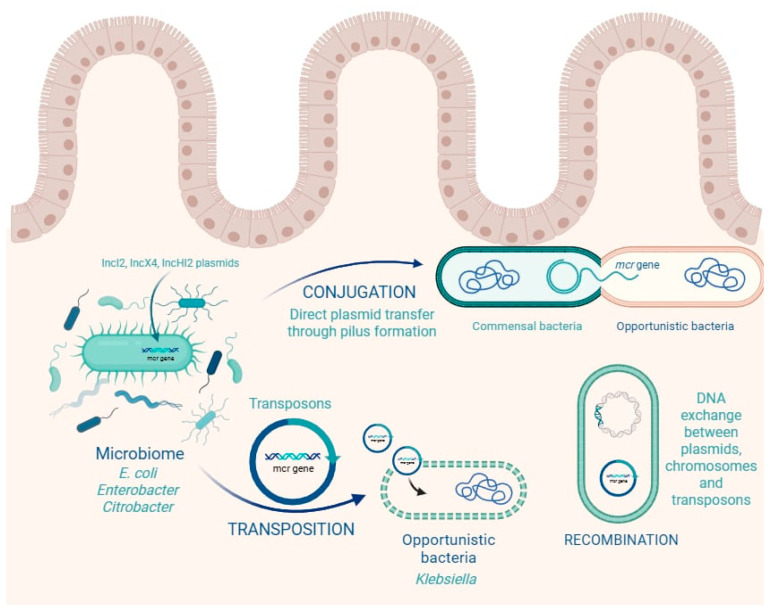
Horizontal transfer of *mcr* genes within the gut microbiota. The gastrointestinal tract provides an ideal environment for plasmid-mediated gene exchange. Commensal bacteria such as *E. coli*, *Enterobacter*, and *Citrobacter* spp. can harbor *mcr*-positive plasmids (e.g., IncI2, IncX4, IncHI2), which are then transferred to pathogenic or opportunistic species such as *K. pneumoniae* during co-colonization. Mobile genetic elements, including insertion sequences (ISApl1) and transposons, facilitate movement of resistance genes across species. Close physical proximity of microbes, biofilm formation, and antibiotic exposure enhance the frequency of conjugation and recombination. This dynamic gene flow highlights the gut as both a reservoir and an amplifier of colistin resistance. Created in BioRender. Pasare, A. (2025) https://BioRender.com/8oungut; Accessed on 31 August 2025.

The human gastrointestinal tract harbors a dense and highly interactive microbial ecosystem that serves not only as a reservoir but also as a dynamic exchange platform for ARGs. Among the most concerning ARGs are the *mcr* genes, which are frequently transmitted via horizontal gene transfer (HGT) mechanisms such as conjugation, transposition, and recombination [11,13] (Figure 2).

Colistin resistance genes, especially *mcr*-1, are typically located on conjugative plasmids (e.g., IncI2, IncX4, IncHI2), insertion sequences (e.g., ISApl1), or transposons that facilitate their movement between bacterial genomes [6]. This genetic mobility allows *mcr* genes to spread rapidly within the gut microbiota, even between phylogenetically distant species. In this context, the gut acts not merely as a passive reservoir but as an active hub of gene exchange under constant evolutionary pressure. The mobilization of *mcr* genes is primarily driven by plasmid-mediated transfer, with IncI2, IncX4, IncHI2, and IncFII plasmids acting as the most important backbones for dissemination [63,64]. These plasmids often carry insertion sequences that facilitate gene mobilization, most notably ISApl1, which is strongly associated with the initial mobilization of *mcr-1* [6], and IS26 or IS903, which contribute to the movement of other *mcr* variants [23,65,66]. Transposons, frequently incorporating insertion sequences at both ends, provide a further mechanism for the capture and relocation of resistance determinants, enabling the clustering of *mcr* with other antimicrobial resistance genes [64]. Integrons and composite transposons enhance this process by promoting recombination and co-selection under antimicrobial pressure [64].

Together, these mobile genetic elements form a modular system in which plasmids serve as vehicles, insertion sequences act as mobilizers, and transposons and integrons expand the genetic context, thereby sustaining horizontal gene transfer and the global spread of *mcr* genes. A key concern is that commensal, non-pathogenic bacteria in the gut—such as *E. coli*, *Enterobacter*, and *Citrobacter* spp.—may acquire *mcr* genes and silently maintain them, only to transfer them to more virulent or opportunistic pathogens during co-colonization events [67]. This possibility was experimentally confirmed in a pediatric study in China, where *mcr*-1-positive *E. coli* isolates from children’s fecal samples successfully transferred colistin resistance to recipient strains in vitro via conjugation [6]. Remarkably, 14 out of 16 donor isolates were able to transfer their plasmids, demonstrating the high transmissibility and stability of these elements [14].

Furthermore, evidence of in vivo interspecies gene transfer is accumulating. Cases where *mcr* genes are simultaneously detected in multiple bacterial species (e.g., *E. coli* and *Citrobacter* spp. from the same fecal sample) strongly suggest active plasmid exchange within the gut environment [35]. These findings are not isolated: similar observations have been made across continents, indicating that HGT is a global phenomenon facilitated by the gut microbiome.

The intestinal environment itself amplifies this risk. Features like biofilm formation, close physical proximity between bacteria, anaerobic conditions, and constant influx of nutrients and antimicrobials create ideal conditions for conjugative plasmid transfer [13,15]. In particular, antibiotic exposure, including to sub-inhibitory levels of colistin—arising either from gut leakage following parenteral dosing in humans or from dietary exposure in veterinary/agricultural settings—has been shown to induce bacterial stress responses, activate SOS pathways, and promote mobilization of plasmids and transposons [67,68].

A compelling example comes from a study in Nigeria, where *mcr*-1, and *mcr*-9 were found on plasmids across multiple Gram-negative genera, including *Klebsiella*, *Enterobacter*, and *E. coli* [35]. All detected *mcr* genes were plasmid-borne, reinforcing the role of mobile elements in shaping the resistome of even healthy individuals. The co-occurrence of different *mcr* genes in the same host or sample highlights the genetic plasticity and evolutionary adaptability of colistin resistance determinants.

Clinically, this means that even in the absence of overt infection, individuals colonized with *mcr*-positive strains may serve as silent disseminators of resistance genes, both within hospitals and in the community. The risk is magnified in ICU patients, immunocompromised hosts, and individuals receiving broad-spectrum antibiotics, where perturbations of the gut flora can further facilitate HGT events and allow *mcr*-carrying pathogens to emerge [34].

Most estimates of plasmid-mediated *mcr* transfer rely on in vitro filter or broth mating assays that may overestimate in vivo rates [69]. These assays typically use high donor-to-recipient ratios, nutrient-rich media, and short incubation times that do not reproduce gut conditions such as anaerobiosis, bile salts, mucus layers, biofilms, nutrient limitation, or immune pressures. Additionally, they underrepresent fitness costs, compensatory evolution, and plasmid incompatibility that shape persistence in the gut [70]. Reported transfer frequencies are further influenced by choices in normalization (per donor, per recipient, or per population), and by selective plates that may favor co-resistant backgrounds [69]. To reduce bias, future studies should report standardized donor-to-recipient ratios, exposure durations, and selective conditions; complement in vitro estimates with gut-on-chip or animal models; and apply culture-independent tools—such as Hi-C–coupled metagenomics, plasmid capture, and strain-resolved assemblies—to quantify in situ gene flow [71,72]. In summary, horizontal gene transfer is a key mechanism by which colistin resistance spreads within the gut microbiota. The combination of mobile genetic elements, commensal reservoirs, and environmental triggers such as antibiotic use ensures that *mcr* genes are not only maintained but actively propagated within the gastrointestinal tract. Addressing this issue requires surveillance strategies that extend beyond clinical pathogens to encompass the entire microbial ecosystem of the human gut.

### 1.4. Early-Life Colonization and Neonatal Implications

The early postnatal period is a critical window in which newborns begin establishing their gut microbiota, a complex community that plays essential roles in immune development, nutrient metabolism, and protection against pathogens. This initial colonization is heavily influenced by maternal microbiota, delivery mode, feeding practices, and hospital environment [73,74]. Unfortunately, mounting evidence shows that antibiotic-resistant organisms—including colistin-resistant Gram-negative bacteria—can also be transmitted to infants during this period.

Of particular concern is the detection of *mcr* genes in neonates within the first days of life. In a multicenter survey of 4907 rectal swabs, *mcr* carriage was 1.0% in mothers (39/3944) and 0.7% in neonates (7/963); 92% (45/49) of *mcr*-positive isolates carried *mcr*-10, with *mcr*-1 (N = 3) and *mcr*-9 (N = 1) also detected, and all neonatal positives were *Enterobacter* spp. carrying *mcr*-10. No mother–infant dyads were concordantly positive, suggesting acquisition via community, vertical, or early environmental routes [35]. The study strongly suggests vertical transmission (intrapartum transfer during delivery—e.g., exposure to maternal vaginal or fecal microbiota)) or early horizontal acquisition from contaminated hospital environments [35,75]. This includes delivery rooms, incubators, feeding equipment, or colonized caregivers.

The same Nigerian study also detected *mcr* genes in maternal fecal samples, providing further evidence for maternal–neonatal transmission pathways [35]. In some cases, *mcr*-positive isolates from neonates were genetically similar to those from their mothers, strengthening the hypothesis of peripartum transmission [14]. Although some neonates in the Nigerian cohort developed sepsis during the study period, the authors report that the *mcr*-positive *Enterobacter* spp. identified from rectal carriage were not the causative pathogens; thus, colonization occurred alongside, rather than as the immediate cause of, neonatal sepsis [35].

Neonates are particularly susceptible to invasive infections because of their immature immune systems, underdeveloped gut barrier, and reduced colonization resistance. If a colistin-resistant strain translocates from the gut to systemic sites, such as the bloodstream or central nervous system, treatment options are severely restricted. Colistin, often the last-resort therapy for multidrug-resistant Gram-negative infections, would be ineffective. Moreover, alternative antibiotics in neonates are limited by safety and pharmacokinetic concerns, particularly in preterm or low-birthweight infants [76].

**Figure 3:**
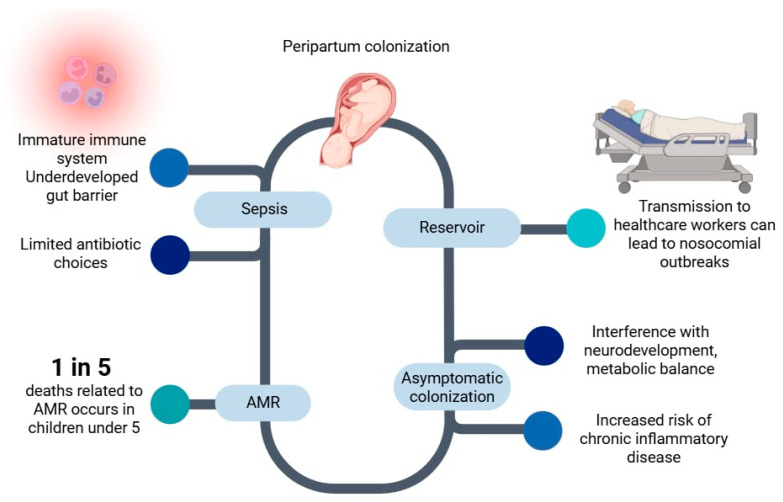
Neonatal gut colonization and antimicrobial resistance risks. Newborns can acquire *mcr*-positive *Enterobacterales* from mothers, hospital environments, or caregivers, often without prior colistin exposure. Their immature immunity, fragile gut barrier, and frequent antibiotic use in NICUs increase the risk of progression from silent carriage to invasive infections (e.g., sepsis). Early colonization may also affect long-term immunity and growth, while colonized neonates can serve as reservoirs for hospital and community transmission. Created in BioRender. Pasare, A. (2025) https://BioRender.com/1a1bmxv; Accessed on 31 August 2025. AMR—antimicrobial resistance.

Even in the absence of infection, asymptomatic colonization with *mcr*-positive bacteria poses long-term risks. Early disruption of the microbiota may interfere with immune programming, metabolic balance, and neurodevelopment [77,78]. Colonization with multidrug-resistant bacteria during the neonatal period has been associated with a higher risk of late-onset sepsis, chronic inflammatory conditions, and growth faltering in early childhood [79] (Figure 3).

The emergence of *mcr*-9 and *mcr*-10 in neonatal gut metagenomes and global isolates signals an escalating risk beyond *mcr*-1. Unlike *mcr*-1, which triggered early global monitoring due to its strong colistin resistance phenotype, *mcr*-9 has spread largely undetected because of its low-level activity. However, the recent emergence of high-expression *mcr*-9 strains that confer full resistance suggests it may follow a more insidious trajectory, silently embedding in clinical and environmental reservoirs before manifesting as a major threat. These dynamics underscore the need for harmonized One Health surveillance to capture both overt and “silent” resistance determinants [80]. Consistently, the identification of *mcr*-9.1 and *mcr*-10.1 in neonatal gut metagenomic datasets further highlights the early-life establishment of these variants, emphasizing the need for harmonized One Health surveillance to capture both overt and “silent” resistance determinants [80,81].

Additionally, colonized neonates can act as reservoirs and transmitters of resistance genes. Transmission to healthcare workers or family members during routine caregiving (e.g., diapering, skin-to-skin contact, or feeding) has been documented [34]. These events can create a cycle of microbial exchange in neonatal intensive care units (NICUs), contributing to nosocomial outbreaks of multidrug-resistant organisms. The role of NICUs as amplification hubs for resistant bacteria is further supported by studies showing that even infants who are not sick may carry pathogens such as *K. pneumoniae* or *E. coli* harboring *mcr* and ESBL genes [82].

The global burden of AMR in neonates is substantial. Children and neonates are highly vulnerable to the impact of antimicrobial resistance, which makes a substantial contribution to morbidity and mortality in this age group; in 2019, an estimated one in five AMR-related deaths occurred in children younger than five years [83]. Many of these infections were previously treatable but are now associated with high mortality due to the emergence of resistance to last-resort antibiotics.

While colistin is rarely administered to neonates because of toxicity concerns, other antibiotics commonly used in NICUs—such as cephalosporins, aminoglycosides, and carbapenems—can disrupt the gut microbiota, leading to loss of protective commensals and the emergence of resistant strains [84]. Furthermore, maternal antibiotic use during labor or cesarean section has been shown to influence neonatal microbiota composition, often favoring the growth of opportunistic pathogens [85].

In summary, early-life colonization with colistin-resistant bacteria represents a serious and under-recognized threat to neonatal health. The presence of *mcr* genes at birth indicates that resistance can breach the earliest biological barriers, even before direct antibiotic exposure. These findings call for urgent interventions, including:

- Routine screening of mothers with recognized risk factors for antimicrobial resistance—such as prior antibiotic exposure, recent hospitalization, or documented carriage of resistant organisms;
- Enhanced hygiene protocols in delivery and NICU environments;
- Minimization of unnecessary antibiotic use in both mothers and neonates; Awareness and education initiatives for clinicians, healthcare workers, and family units regarding AMR and colistin resistance, especially in neonatal intensive care settings.

Protecting the neonatal microbiome is essential not only for immediate infection control but also for long-term health outcomes spanning immunity, metabolism, and development. In a cohort study of 34 very-low-birth-weight, human-milk-fed preterm infants, Kiu et al. employed genome-resolved shotgun metagenomics and targeted culturomics to elucidate the combined impact of probiotics and antibiotics on the developing gut microbiome and resistome [86]. Infants supplemented with a probiotic mixture (*Bifidobacterium bifidum* and *Lactobacillus acidophilus*) demonstrated a pronounced reduction in antibiotic resistance gene prevalence and MDR pathogen load compared to non-supplemented controls. Their gut microbial profiles resembled those of full-term infants, exhibiting enriched beneficial genera such as *Bifidobacterium* and attenuated colonization by potential pathobionts including *Enterococcus*, *Klebsiella*, and *Escherichia*. Notably, despite these positive shifts, MDR *Enterococcus* strains with high horizontal gene transfer potential persisted, highlighting the necessity for ongoing surveillance and nuanced interventions in neonatal units. These findings demonstrate the capacity of probiotic supplementation not only to restore microbial balance but also to reduce the neonatal resistome footprint under antibiotic pressure [86].

Despite the clinical significance of early colonization with *mcr*-positive bacteria, neonatal-specific diagnostics and interventions remain underdeveloped. There are no standardized screening protocols for *mcr* carriage in neonates, and current NICU infection control practices typically overlook colistin resistance [82]. Furthermore, microbiota-directed therapies—including FMT, probiotics, or Clustered Regularly Interspaced Short Palindromic Repeats (CRISPR)-based interventions—have not been systematically studied in neonatal populations, particularly in preterm or low-birthweight infants [84,87]. This research gap limits opportunities for early intervention and highlights the need for targeted clinical trials and policy development in this vulnerable group.

Emerging evidence suggests that neonatal gut dysbiosis—disrupted early microbial colonization—can have enduring health implications beyond infection risk. Prospective cohort studies of preterm infants show that early-life microbial imbalances are associated with neurological and behavioral trajectories by age 4, including associations of taxa like *Veillonella dispar* and *Enterobacterales* with diagnoses such as ADHD and autism spectrum behaviors [88]. Separately, narrative reviews highlight that early-life gut dysbiosis substantially raises the risk of later metabolic disorders, including obesity, type 1 diabetes, allergic diseases, cardiovascular conditions, and neurological sequelae, reflecting the Developmental Origins of Health and Disease paradigm [89]. These results bring attention to the importance of neonatal resistome surveillance not only for antimicrobial resistance but also in understanding how *mcr*-mediated alterations may contribute to broader immunometabolic programming across the lifespan.

### 1.5. Clinical and Public Health Risks of Gut Carriage of Colistin-Resistant Enterobacterales

Gut colonization with colistin-resistant *Enterobacterales* represents a serious dual threat, affecting both individual carriers and broader public health systems. These organisms, especially those harboring *mcr* genes, are increasingly reported in both community and healthcare settings, often embedded within complex MDR plasmids [6].

#### 1.5.1. Clinical Risks

For the individual carrier, gut colonization with *mcr*-positive strains—such as *E. coli*, *K. pneumoniae*, or *Enterobacter* spp.—poses a significant clinical risk. These organisms may act as a source of endogenous infection, including urinary tract infections, intra-abdominal abscesses, and pneumonia, or bacteremia, particularly in hospitalized or immunocompromised individuals [34]. Since colistin is often reserved as a last-resort antibiotic against MDR Gram-negative pathogens, infections caused by *mcr*-harboring organisms may be virtually untreatable if resistance extends to other new drug classes [90].

Compounding this threat, *mcr* genes are frequently co-located with extended-spectrum β-lactamase (ESBL) or carbapenemase genes (e.g., *bla_CTX-M_. bla_NDM_*), rendering the strains resistant to multiple or all available treatment options [14]. Clinical outcomes in such cases are poor, with higher rates of treatment failure, ICU admission, and high mortality rates [91].

Colonization is also a predictive risk factor for future infection. In hospitalized patients, especially those undergoing surgery or invasive procedures, colonization with colistin-resistant organisms significantly increases the risk of subsequent infection by the same strain [92]. Thus, gut carriage silently sets the stage for life-threatening infections in vulnerable patients.

#### 1.5.2. Public Health Risks

From a public health standpoint, individuals colonized with *mcr*-positive bacteria serve as reservoirs and vectors of resistance. Shedding of these organisms in feces can lead to environmental contamination in hospitals, households, and public spaces, particularly where hygiene infrastructure is poor. Studies have identified *mcr*-positive *E. coli* on hospital surfaces, implicating fecal transmission as a source [93].

Colonized individuals may unknowingly spread resistant strains to family members, healthcare workers, or community contacts via the fecal–oral route—similar to the global dissemination pattern of ESBL-producing *E. coli* [94]. International travel and global food trade exacerbate this problem. Travelers to countries with high agricultural use of colistin have been shown to acquire *mcr*-positive strains during brief trips, with higher carriage rates documented among returning European travelers [95].

In some resource-limited settings, community carriage rates are alarmingly high. Studies from Bolivia and Vietnam have reported *mcr* gene prevalence in gut flora reaching 38% and over 60%, respectively [96,97]. These individuals act as silent amplifiers of resistance within the population, often lacking access to proper sanitation or microbiological testing.

The food supply is another significant route of transmission. *mcr*-positive *E. coli* and *Salmonella* have been isolated from retail poultry and pork products in Europe and Asia. One survey detected *mcr*-harboring strains in up to 70% of retail poultry samples, illustrating a direct pathway for resistance genes to enter the human gut via food [38]. This cycle—involving humans, animals, and the environment—has turned colistin resistance into a global ecological threat. The worst-case scenario is the convergence of *mcr* with carbapenem-resistance genes in a single strain, resulting in pan-resistant *Enterobacterales* against which no effective antibiotics remain [9].

A 2025 systematic review on ARG detection shows that health risk estimates depend not only on how common these genes are but also on the methods used to find them. Curated databases such as the Comprehensive Antibiotic Resistance Database and the National Database of Antibiotic Resistant Organisms mainly capture well-known genes, whereas machine learning-based tools like DeepARG and the Hierarchical Multi-task Deep-learning ARG framework (HMD-ARG) can reveal novel or rare ones [98]. Because these resources differ in scope and accuracy, reliance on a single or outdated approach may underestimate resistance, especially in low-resource or environmental settings. By combining curated and predictive methods, the review provides updated risk assessments that highlight the gut microbiome as a central hub for ARG spread and stress the need for standardized One Health–aligned surveillance to improve global AMR projections [98]. In summary, gut carriage of colistin-resistant *Enterobacterales* is more than a passive finding; it is a clinical time bomb. For the carrier, it may presage an untreatable infection. For society, it accelerates the spread of last-resort antibiotic resistance, undermining both hospital infection control efforts and community-level public health. Active surveillance, improved diagnostic tools, antibiotic stewardship, and infection prevention strategies must be implemented to prevent this silent threat from escalating further.

### 1.6. Alternative Strategies to Colistin for Managing Multidrug-Resistant Enterobacterales

As resistance to colistin increases due to the dissemination of *mcr* genes, the clinical need for effective alternatives has become critical. Emerging and existing alternatives include novel β-lactam/β-lactamase inhibitor combinations, cefiderocol, bacteriophage therapy, antimicrobial peptides, and CRISPR-based antimicrobials. These approaches vary in readiness from approved therapeutics to experimental solutions and collectively represent a multifaceted response to the challenge of colistin resistance.

Recent years have seen the development of several antibiotics capable of treating infections caused by carbapenem-resistant and colistin-resistant *Enterobacterales*. Notable agents include:

- Cefiderocol, a siderophore cephalosporin with potent activity against Gram-negative pathogens, including *mcr*-positive strains [99].
- Ceftazidime-avibactam and meropenem-vaborbactam, which are active against KPC-producing organisms, although their efficacy may be limited in *mcr*-co-harboring isolates [92].
- Tigecycline and eravacycline (new-generation tetracyclines) often retain activity against colistin-resistant isolates and are used for intra-abdominal and complicated soft tissue infections [100].
- Fosfomycin has also shown synergistic potential in combination regimens, especially for urinary tract infections [101].

These agents are not without limitations (e.g., reduced efficacy in bloodstream infections or rapid emergence of resistance), but they expand the therapeutic arsenal beyond colistin. Notably, treatment-emergent cefiderocol resistance has been documented: across phase 3 trials (APEKS-NP, CREDIBLE-CR), >4-fold MIC increases occurred in ~7% of treated patients, though only ~1–2% crossed clinical resistance breakpoints. Observational series and case reports have further described sporadic emergence (e.g., in *A. baumannii*, *E. cloacae*, and *E. coli*), typically under high bacterial burden, prolonged exposure, or suboptimal source control [102,103,104,105].

Antimicrobial peptides (AMPs), such as defensins and synthetic analogs, act by disrupting bacterial membranes and can retain activity despite *mcr*-mediated resistance [106]. Synthetic AMPs and peptidomimetics are being designed to overcome issues of toxicity and stability. Preclinical studies demonstrate their ability to kill colistin-resistant bacteria and suppress resistance evolution [107].

### 1.7. Decolonization Strategies for *mcr*-Positive Gut Colonization

**Figure 4:**
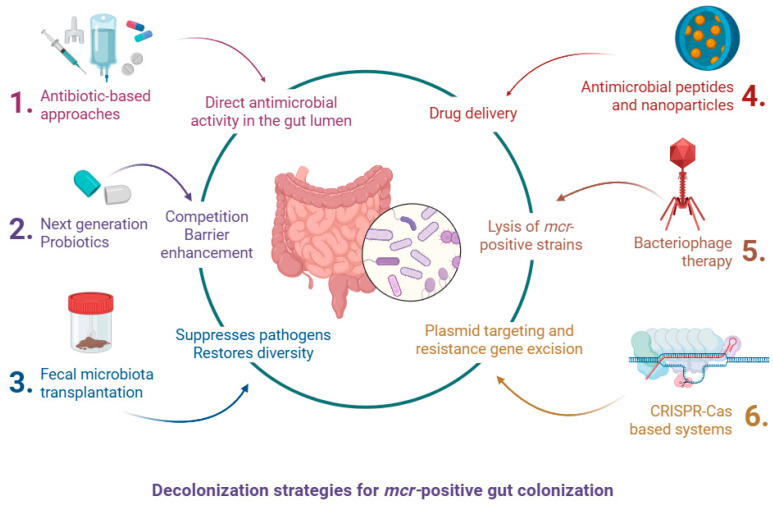
Strategies under investigation to eradicate *mcr*-positive *Enterobacterales* from the gut. Approaches include non-absorbable antibiotics, fecal microbiota transplantation to restore colonization resistance, bacteriophage therapy targeting resistant strains, CRISPR-Cas systems to selectively remove resistance genes, probiotics/next-generation biotherapeutics to rebalance the microbiota, and antimicrobial peptides or nanoparticles with activity against *mcr*-mediated resistance. These interventions are promising but remain largely experimental. Created in BioRender. Pasare, A. (2025) https://BioRender.com/ml031w1; Accessed on 31 August 2025.

The increasing prevalence of *mcr*-positive *Enterobacterales* in the human gastrointestinal tract represents a major challenge for infection control. Gut colonization often precedes invasive infections and facilitates horizontal transmission of resistance genes. Although no standard treatment currently exists for decolonizing the gut from *mcr*-harboring organisms, several investigational strategies have shown promise. These approaches range from conventional antimicrobials to next-generation microbiome-based and gene-targeted interventions (Figure 4).

#### 1.7.1. Antibiotic-Based Approaches

One of the earliest approaches explored for decolonization involved the use of non-absorbable oral antibiotics. Agents such as colistin, rifaximin, aminoglycosides (e.g., neomycin, gentamicin), and their combinations have been trialed to reduce intestinal colonization with multidrug-resistant *Enterobacterales* (MDRE). In small studies, oral gentamicin or colistin combined with rifaximin showed transient reductions in colonization density but failed to eradicate the resistant strains [108,109]. Furthermore, these regimens pose significant risks: they can further disrupt the gut microbiota, select for resistant subpopulations, and may co-select for resistance genes on shared plasmids [109]. In the case of *mcr*-positive bacteria, the utility of oral colistin is inherently limited, as the resistance gene renders the target ineffective.

Overall, antibiotic-based decolonization strategies remain low in feasibility and clinical readiness. While they are conceptually straightforward and relatively inexpensive, their usefulness is undermined by high relapse rates, disruption of commensal gut flora, and the risk of selecting for additional resistance mechanisms. No standardized protocols have been established, and the few available trials are small and heterogeneous. Consequently, antibiotic-based approaches are unlikely to provide a sustainable or widely applicable solution for *mcr*-positive gut carriage.

#### 1.7.2. Fecal Microbiota Transplantation (FMT)

FMT involves the transfer of stool from a healthy donor into the gastrointestinal tract of a colonized individual to restore microbial diversity and suppress pathogenic organisms. It is now a well-established treatment for recurrent *Clostridioides difficile* infection and has shown efficacy in decolonizing MDRE [110,111]. Although limited data exist specifically on *mcr*-positive bacteria, a case series reported partial success in eradicating intestinal carriage of *mcr*-1-positive *E. coli* after repeated FMTs [87]. Mechanistically, FMT restores colonization resistance, introduces competitive taxa, and can reduce inflammation and gut permeability, thereby reducing opportunities for resistant organisms to persist. Challenges include donor screening, regulatory oversight, and the variability in clinical responses, but ongoing trials are investigating its potential in AMR decolonization.

While FMT has shown promise in decolonizing multidrug-resistant pathogens, robust clinical data specifically addressing *mcr*-positive *Enterobacterales* remain limited. Most available evidence stems from isolated case series or anecdotal reports, with no randomized controlled trials currently available to guide standardized use in this context [87,111]. Additionally, challenges such as donor variability, regulatory constraints, and potential risks in immunocompromised patients further complicate its broader adoption [110]. This highlights a critical need for prospective, multicenter trials focused on microbiome-based decolonization strategies for colistin-resistant organisms.

Another limitation is the variability in outcomes between different centers and protocols, which makes it difficult to compare results or define standardized success rates. The long-term durability of decolonization following FMT is also uncertain, as relapse or recolonization may occur, particularly in settings with high environmental or household exposure to resistant bacteria. In addition, the optimal route of administration (oral capsules, colonoscopy, or nasogastric delivery) has not been established for resistant *Enterobacterales*, and each carries distinct logistical and safety considerations. Finally, the cost and complexity of donor screening and material preparation restrict scalability, especially in low-resource regions where *mcr* carriage is often most prevalent [112,113,114].

#### 1.7.3. Bacteriophage Therapy

Bacteriophage (phage) therapy has re-emerged as a promising alternative or adjunct to antibiotics for targeting multidrug-resistant organisms, including colistin-resistant *Enterobacterales*. Phage therapy uses lytic viruses that specifically infect and kill bacteria. It offers a highly selective and adaptable strategy to target *mcr*-positive strains without disturbing the broader microbiome. In vitro and in vivo models have demonstrated that bacteriophages can reduce the burden of colistin-resistant *E. coli* and *K. pneumoniae* [115]. Certain naturally occurring or engineered bacteriophages have demonstrated synergistic activity with colistin, including against *mcr*-1-producing strains, by increasing bacterial membrane permeability or disrupting resistance pathway [116,117]. However, phage resistance, narrow host ranges, and limited regulatory pathways remain obstacles. Personalized phage cocktails, adjusted to the colonizing strain’s genotype, may offer future solutions for selective gut decolonization, particularly in high-risk groups such as immunocompromised patients, neonates, or those with severe comorbidities.

Several in vivo and ex vivo studies have demonstrated that lytic phages can significantly reduce intestinal loads of *mcr*-positive *E. coli* and *K. pneumoniae*, particularly when administered orally or via encapsulated delivery systems to withstand gastric acid [118,119]. Phage cocktails have been shown to prevent the emergence of resistance more effectively than monophage treatments and may synergize with antibiotics like colistin or tigecycline [120]. Additionally, engineered or synthetic phages designed to express CRISPR-Cas systems or biofilm-degrading enzymes have shown enhanced efficacy against biofilm-forming, colistin-resistant strains [121].

Despite encouraging laboratory and animal data, the clinical feasibility of phage therapy for gut decolonization remains uncertain. Host range limitations require personalized cocktails matched to individual colonizing strains, a process that is time-consuming and not readily scalable. Resistance to phages can emerge rapidly, particularly in the gut where high bacterial densities promote adaptation. Regulatory pathways for phage use differ widely between countries, and standardized manufacturing practices are not yet established. To date, clinical use has been largely confined to compassionate-use cases or early-phase trials, placing phage therapy at a low-to-intermediate readiness level. Its future utility will depend on overcoming these technical, regulatory, and scalability barriers [122,123,124].

#### 1.7.4. CRISPR-Cas-Based Systems

CRISPR-Cas systems, originally developed for gene editing, can be reprogrammed to target and eliminate specific DNA sequences such as resistance genes. Experimental models have used CRISPR-Cas constructs to selectively excise *mcr* plasmids from *E. coli* without affecting host cell viability or the commensal microbiota [125].

These systems can be delivered via engineered bacteriophages or conjugative plasmids, enabling them to spread across bacterial populations in the gut [126]. While still in early research phases, CRISPR-Cas antimicrobials represent a groundbreaking approach that could allow for precise, resistance-gene-specific decolonization, minimizing ecological disruption.

Although CRISPR-Cas offers exceptional specificity in targeting and removing *mcr*-mediated resistance, practical implementation faces substantial technical and translational hurdles. First, the delivery of CRISPR constructs into the complex gut microbiome remains inefficient. While vectors such as conjugative plasmids, bacteriophages, and nanoparticles have been explored, each has significant limitations in terms of efficiency and stability in vivo [127,128].

Several innovative approaches have demonstrated promise. For instance, engineered conjugative systems achieved over 99.9% elimination of target antibiotic-resistant *E. coli* in the mouse gut—highlighting the potential of horizontal transfer vehicles embopress.org. Additionally, phage-mediated delivery systems have been shown to reach specific bacterial strains within the murine gastrointestinal tract, yet efficiency and host range remain major constraints [129].

Furthermore, resistance to CRISPR-Cas interventions can emerge rapidly. Escape mechanisms include spontaneous mutations in Cas genes or target protospacers, spacer deletion via homologous recombination, and the presence of anti-CRISPR proteins in target bacteria [128]. On the regulatory and safety fronts, deployment of CRISPR-Cas within live microbial ecosystems raises complex biosafety issues. Off-target effects could disrupt commensal populations or facilitate horizontal transfer of gene-editing constructs. To date, regulatory pathways for such “live-edit genetic interventions” are undefined in most jurisdictions.

In summary, while CRISPR-Cas antimicrobials are at an exciting proof-of-concept stage, current readiness remains low. Overcoming key barriers—effective in situ delivery, resistance mitigation, and biosafety/regulatory frameworks—is essential before these methods can translate into viable clinical tools for decolonizing *mcr*-positive gut reservoirs [130,131].

#### 1.7.5. Probiotics and Microbiota Modulators

Probiotic interventions aim to shift the gut microbial composition by introducing beneficial bacterial strains that can compete with resistant pathogens. *Lactobacillus* and *Bifidobacterium* species have been shown to inhibit the adhesion and colonization of MDRE in vitro and in animal models [132,133,134]. However, evidence of efficacy against *mcr*-positive *Enterobacterales* is sparse.

Recent studies suggest that probiotics and microbiota-directed therapies may offer promising adjunct strategies for reducing intestinal colonization by multidrug-resistant organisms, including colistin-resistant *Enterobacterales*. In a pilot study among long-term care residents, a multispecies probiotic intervention was associated with a transient reduction in MDRE carriage, including colistin-resistant strains, and promoted increased microbial diversity [135]. While complete eradication was not achieved, the probiotic effect was more pronounced in individuals with higher baseline colonization, indicating possible niche competition dynamics. Similarly, targeted microbiota restoration using biotherapeutic formulations significantly decreased intestinal loads of ESBL-producing and colistin-resistant *Enterobacterales*, without disrupting commensal flora, supporting the viability of non-antibiotic decolonization approaches [136]. Beyond conventional probiotics, next-generation candidates such as *Akkermansia muciniphila* are drawing attention due to their ability to enhance epithelial barrier integrity and modulate host immune responses. Preclinical studies have shown that *A. muciniphila* can reinforce the mucus layer, promote regulatory T-cell activity, and create metabolic environments unfavorable to pathobiont expansion [137,138]. Furthermore, this strain has been linked to upregulation of tight junction proteins and modulation of IL-22 signaling, suggesting not only the gut microbiota’s role as a reservoir of colistin resistance, but also its potential as a therapeutic target for restoring colonization resistance against multidrug-resistant organisms [139].

Although probiotics and next-generation biotherapeutics represent a safe and conceptually scalable approach, their clinical readiness for eradicating *mcr*-positive *Enterobacterales* is low. Conventional probiotics such as *Lactobacillus* and *Bifidobacterium* have been evaluated in randomized clinical trials, but outcomes for decolonizing multidrug-resistant *Enterobacterales* have been inconsistent, often showing only transient effects that diminish once supplementation ceases [140,141,142]. Results also vary substantially between individuals depending on baseline microbiome composition and host factors [143]. Next-generation candidates such as *Akkermansia muciniphila* and *Faecalibacterium prausnitzii* demonstrate promising effects in restoring colonization resistance and improving host metabolism, but their culture requirements, formulation stability, and safety in vulnerable populations remain significant barriers [144]. Regulatory approval of live biotherapeutics requires rigorous demonstration of safety, batch-to-batch consistency, and long-term efficacy.

While progress has been made in defining regulatory frameworks, including FDA guidance on chemistry, manufacturing, and control standards, current requirements remain challenging to meet for next-generation products [145]. Trials to date suffer from lack of standardization in probiotic strains, dosing regimens, and participant selection [146]. Moreover, reported outcomes are often inconsistent between studies and individuals, complicating interpretation and application [147]. Critically, definitive data—particularly randomized controlled trials demonstrating sustained reduction in *mcr*-positive colonization—are still lacking. At present, probiotics and next-generation biotherapeutics should be considered experimental adjuncts rather than practical clinical tools for decolonizing *mcr*-positive gut reservoirs.

#### 1.7.6. Targeted Antimicrobial Peptides and Nanoparticles

Recent advances in antimicrobial delivery systems have led to the development of targeted peptides and nanoparticles designed to overcome outer membrane resistance mechanisms conferred by *mcr* genes. These include cationic peptides that mimic host defense peptides, antimicrobial-loaded liposomes, and silver or polymer-based nanoparticles that can penetrate bacterial envelopes and deliver localized bactericidal effects [148]. Some formulations show activity even in the presence of *mcr*-mediated lipid A modifications. Though primarily tested in vitro and in animal models, these technologies may eventually allow targeted eradication of resistant strains in the gut while preserving overall microbial diversity.

Beyond biological approaches, metabolic adjuncts are also emerging. For instance, vitamin B6 has been shown to resensitize *mcr*-harboring Gram-negative bacteria to colistin in preclinical models, offering a safe and potentially scalable adjunct to conventional therapies [149].

Antimicrobial peptides (AMPs) and nanoparticle-based agents are attractive alternatives because of their broad-spectrum activity and potential to disrupt biofilms, which could make them useful in clearing multidrug-resistant *Enterobacterales*. However, most data are preclinical, and several challenges limit their feasibility. Many AMPs exhibit poor stability in the gastrointestinal tract due to proteolytic degradation and variable pH, which reduces their activity in vivo [150]. Cytotoxicity and immunogenicity also remain concerns, with some peptides inducing host cell damage at therapeutic concentrations [151]. Similarly, nanoparticles such as silver, chitosan, and lipid-based carriers have shown in vitro bactericidal activity and synergy with antibiotics, but safety issues related to accumulation, oxidative stress, and off-target toxicity restrict their translation [152]. To date, no AMP or nanoparticle strategy has been validated in controlled human trials for decolonizing *mcr*-positive organisms, placing their readiness level at an early preclinical stage.

### 1.8. Artificial Intelligence–Driven Prediction of *mcr* Emergence

Recent advances in artificial intelligence (AI) and machine learning (ML) are opening new opportunities to anticipate the emergence and dissemination of *mcr* genes across microbial populations. Traditional surveillance relies on retrospective sequencing and culture-based detection, which often lags behind real-time resistance dynamics. In contrast, AI models trained on bacterial genomic and metagenomic datasets can identify sequence motifs, mobile genetic elements, or plasmid signatures linked to *mcr* mobilization, enabling early detection before high-level resistance evolves. Reviews highlight the application of ML in predicting antimicrobial resistance patterns using whole-genome data across diverse pathogens, including early efforts employing classical methods like decision trees and k-mer analysis to forecast novel resistance determinants [153,154,155,156].

Innovative frameworks such as evolutionary accumulation modeling (EvAM) apply ML to reconstruct and predict MDR gene acquisition trajectories without direct longitudinal sampling. By modeling evolutionary “steps” in resistance development, EvAM provides a probabilistic view of how novel resistance determinants like *mcr* variants may emerge and spread [157]. Advances in genomic language modeling—employing transformer-based architectures to capture both sequence context and the related literature—hold promise for classifying resistance gene classes and enhancing predictive accuracy for emergent *mcr* subtypes [58,158]. Multimodal AI models that integrate molecular structures, genomic contexts, and deep learning—such as contrastive learning frameworks (e.g., CL-MFAP)—can accelerate antibiotic discovery and potentially forecast resistance gene–drug interactions, creating complementary tools to *mcr* surveillance [159].

At the same time, translational challenges remain. Current ML pipelines often treat genes as independent predictors, disregarding structural or functional linkages, and their generalizability is frequently limited by population structure, class imbalance, and heterogeneous laboratory phenotypes. Furthermore, clinical uptake has been limited, with only a small number of methods implemented in practice within healthcare or public health contexts, and the need for interpretability remains a critical requirement to ensure end-user confidence [160,161].

Collectively, these AI-driven approaches offer a future roadmap for integrating computational biology with microbiological surveillance, helping anticipate and forestall the rise of *mcr*-mediated resistance through predictive monitoring and strategic intervention.

### 1.9. Antimicrobial Stewardship and Infection Control Implications

To effectively address the rising threat of colistin resistance in the gut microbiota, robust antimicrobial stewardship and comprehensive infection control strategies must be implemented across clinical, agricultural, and public health domains. The use of colistin in human medicine should be limited strictly to last-resort scenarios involving life-threatening infections caused by multidrug-resistant Gram-negative pathogens. Its application must be governed by clear clinical guidelines and oversight by infectious disease specialists. Evidence suggests that inappropriate or sub-therapeutic use of colistin exerts selective pressure on gut flora, encouraging the emergence and persistence of *mcr*-positive bacteria. Sub-inhibitory concentrations, in particular, have been linked to enhanced plasmid transfer and resistance propagation. Therefore, careful consideration of dosage, therapeutic monitoring, and exploration of alternative treatments—such as novel antimicrobials, phage therapy, or antimicrobial peptides—is warranted to preserve colistin’s efficacy [6,162,163,164,165].

In parallel, there is an urgent need to eliminate colistin’s use in food animal production. The widespread application of colistin as a growth promoter and prophylactic agent in livestock has been a key factor in the global dissemination of *mcr* genes. Regulatory action taken by several countries, including China and members of the European Union, to ban or restrict colistin use in agriculture has already shown measurable impact in reducing *mcr* prevalence in both animals and humans. However, many regions still lack enforceable policies, and the export of veterinary-grade colistin to low- and middle-income countries remains problematic. A globally coordinated regulatory framework—integrated through a One Health lens—should bring together stakeholders from human health, veterinary medicine, and agriculture to abolish non-therapeutic colistin use and curtail resistance at its environmental source [8,14,166,167]. The broader strengths, weaknesses, opportunities, and threats of such One Health interventions are summarized in Table 2.

Surveillance of colistin resistance must be expanded and refined. Targeted screening programs in hospitals and communities are essential to detect colonization early and prevent outbreaks. High-risk populations—such as ICU patients receiving multiple antibiotics, neonates in intensive care, and individuals transferred from regions with high *mcr* prevalence—should be prioritized for screening. Surveillance should not be limited to clinical isolates alone; environmental monitoring of sewage, wastewater, and farm runoff can provide early warning of resistance hotspots. Importantly, diagnostic laboratories must incorporate reliable colistin susceptibility testing into their protocols, using broth microdilution or other validated methods to ensure detection of *mcr*-mediated resistance, which may otherwise go unnoticed due to technical challenges in routine antimicrobial testing [35,52,93,168]. However, significant barriers remain: broth microdilution testing is technically demanding and resource-intensive; many low- and middle-income countries lack laboratory capacity and surveillance infrastructure; standardized protocols across human, veterinary, and environmental sectors are still limited; and data sharing between local, national, and international networks is often fragmented [169].

The data reveal substantial geographic variability in *mcr* distribution, with particularly high rates in Asian poultry systems (e.g., Bangladesh, China), widespread environmental detection in Europe and Asia, and emerging clinical cases in Africa, South America, and Eastern Europe [170,171,172,173,174,175,176,177,178,179,180,181]. Notably, *mcr*-1 remains the most dominant variant globally, often co-occurring with multidrug-resistant profiles [41]. In Romania, recent clinical reports have documented the first human isolates carrying plasmid-mediated *mcr*-1, while earlier studies had already detected *mcr*-1 in poultry-associated *E. coli*, highlighting the persistence and zoonotic potential of this resistance gene [174,175].

Infection control efforts within healthcare facilities must be intensified to prevent horizontal transmission of colistin-resistant organisms. Patients known to harbor *mcr*-positive *Enterobacterales*—especially those co-carrying ESBL or carbapenemase genes—should be managed using strict contact precautions, including gloves, gowns, and possibly single-room isolation. These measures are particularly critical in settings such as intensive care units and neonatal wards. Environmental decontamination protocols must be optimized to eliminate Gram-negative pathogens from surfaces that may become contaminated via fecal shedding. Coordination between infection prevention teams and antimicrobial stewardship units is vital to identify high-risk patients, initiate screening where needed, and ensure compliance with protocols designed to minimize transmission within the hospital environment [182,183].

Future efforts should integrate cutting-edge bioinformatics and economic modeling to enhance the translational impact of resistome research. Advances in shotgun metagenomic sequencing, especially approaches enabling species-level resolution of resistance genes (e.g., Argo, a long-read overlapping tool for precise ARG quantification) significantly elevate the capacity to track *mcr* dissemination in both clinical and environmental reservoirs [184,185,186]. Meanwhile, recent modeling studies estimate that antibiotic-resistant infections in 2019 alone cost approximately US $693 billion in hospital care and US $194 billion in productivity losses, underscoring the staggering economic burden of AMR [187]. By combining high-resolution resistome profiling with robust economic impact assessments, policymakers can better prioritize interventions and allocate resources to curb *mcr*-mediated resistance within a One Health framework.

Altogether, addressing colistin resistance in the gut microbiota demands a multidimensional response rooted in stewardship, surveillance, and infection control. Limiting colistin use to absolute clinical necessity, eradicating agricultural misuse, strengthening laboratory and environmental surveillance, and enforcing rigorous hygiene practices are the foundational pillars of such a response. Gut microbiota alterations, particularly the depletion of short-chain fatty acids-producing bacteria and rise in opportunistic pathogens, have been linked to heightened systemic inflammation and cytokine responses [133]. Only by acknowledging the interconnected nature of human, animal, and environmental health can the global community mount an effective defense against the further spread of colistin resistance. The convergence of antimicrobial stewardship and infection prevention, aligned through a One Health framework, is essential to preserve this critical last-line antibiotic and mitigate the risks posed by *mcr*-positive bacteria in the gut ecosystem [62,188].

## 2. Conclusions

The gut microbiota has emerged as a central player in the propagation of colistin resistance, acting not only as a reservoir for *mcr*-positive organisms but also as a dynamic environment where resistance genes are exchanged and stabilized. The silent colonization of the gastrointestinal tract by colistin-resistant *Enterobacterales*—often in individuals without prior antibiotic exposure—illustrates how resistance can spread undetected within communities and across species boundaries.

This microbial ecosystem is particularly vulnerable to selective pressures from antibiotic use in both human medicine and agriculture. Practices such as routine colistin administration in livestock have contributed to the enrichment of resistant strains in the gut, with implications for both foodborne transmission and environmental dissemination. Meanwhile, the persistence of *mcr* genes in commensals poses a risk of horizontal gene transfer to pathogenic bacteria, particularly in hospitalized or immunocompromised patients.

Addressing colistin resistance at its microbial source will require more than antimicrobial stewardship—it demands targeted strategies to monitor, disrupt, and ultimately reduce carriage within the gut. This includes investment in microbiome-based interventions, improved diagnostics for colonization detection, and enhanced surveillance capable of capturing gut-level resistance dynamics. Particular attention must be paid to high-risk populations, such as neonates, where early colonization may have long-term clinical consequences.

By prioritizing the gut microbiota in both research and policy, and aligning efforts across human, animal, and environmental health sectors, it is still possible to limit the spread of *mcr*-mediated resistance and preserve the therapeutic value of colistin in the face of rising antimicrobial threats. Recent advances in AI- and ML-based genomic surveillance further provide a roadmap for anticipating and mitigating the emergence of *mcr*-mediated resistance, complementing traditional retrospective approaches.

## Figures and Tables

**Table 1:** Summary of *mcr* gene variants, bacterial hosts, and associated plasmid types.

Gene	Year and Country of First Report	Typical Hosts and Sources	Plasmid/Location	Horizontal Transfer Efficiency	Colistin MIC Impact	Notes
*mcr*-1	2015, China (*E. coli* from pigs and meat)	*E. coli*, *Klebsiella* *Enterobacterales*	IncI2, IncX4, IncHI2	High—highly conjugative on diverse plasmid backbone widely disseminated	2–8 mg/L [17]	Most globally prevalent; food/clinical link
*mcr*-2	2016, Belgium (pigs)	*E. coli*	IncX4	Efficient but less prevalent	Similar to *mcr-*1, commonly 4–8 mg/L in *E. coli* [18,19]	Limited global spread
*mcr*-3	2017, China (pig feces)	*E. coli*, *Salmonella*, *Aeromonas*	IncHI2, IncP, IncFII	Moderate, some chromosomal insertions	4–8 mg/L in *E. coli*, may be higher in other hosts (e.g., *Aeromonas*) [20,21]	Environmental reservoirs important
*mcr*-4	2017, Italy (swine feces)	*Salmonella enterica*, *E. coli*	IncHI2, ColE-like	Transferable	4–8 mg/L [22]	Sporadic
*mcr*-5	2017, Germany (animal feces, food)	*Salmonella*, *E. coli*	IncX1, chromosomal	Integrated into chromosomes; conjugation possible but less efficient.	~8 mg/L in *Salmonella Paratyphi B* ~4 mg/L in other isolates [23]	Detected mostly in food chain
*mcr-*6	2017, UK (pig)	*Moraxella* spp.	Chromosomal	Not plasmid-borne Chromosomally located in *Moraxella*; no evidence of efficient conjugative transfer.	Low–moderate (1–2 mg/L) [24]	Rare
*mcr*-7	2018, China (chicken)	*K. pneumoniae*	IncI1	Transferable Experimentally shown to mobilize between *Enterobacterales*.	4–8 mg/L [25]	Limited distribution
*mcr*-8	2018, China (cattle, humans)	*K. pneumoniae*, *Raoultella*	IncFII(K), IncHI2	Efficient in *K. pneumoniae* Demonstrated conjugation	8–16 mg/L in *K. pneumoniae*; 16 mg/L have been reported for *mcr*-8.1 [26,27]	Clinical outbreaks in Asia
*mcr-*9	2019, USA (human/animal/food/environment)	*Serratia*, *Morganella*	IncHI2, IncFII	Conjugative, inducible (qseB/qseC regulation)	frequently “silent” with MIC ≤ 2 mg/L unless induced; upon induction, MIC may increase [28,29]	Widespread, but variable phenotypic resistance
*mcr*-10	2020, China (clinical *Enterobacter roggenkampii*)	*Enterobacter* spp., *Klebsiella*	IncFII(K)	Transferable Conjugation demonstrated	~4 mg/L (*E. roggenkampii*), but broader ranges from 4 up to >16–128 mg/L in *Enterobacter* spp.; inducibility has been observed [30,31]	Emerging, low prevalence

MIC—minimum inhibitory concentration.

**Table 2:** SWOT analysis of One Health interventions targeting *mcr*-mediated resistance.

Strengths	Weaknesses	Opportunities	Threats
Established AMR surveillance platforms (e.g., GLASS, national veterinary monitoring programs) provide a ready framework into which *mcr* surveillance can be embedded across clinical, veterinary, and environmental compartments.	Sparse randomized controlled trials for decolonization strategies; heterogeneous designs limiting inference	Incorporation of wastewater-based metagenomics and high-throughput sequencing pipelines could enable near real-time monitoring of *mcr* gene circulation, improving early detection of resistance hotspots.	Co-selection pressures from non-polymyxin antibiotics, disinfectants, and heavy metals sustaining *mcr* despite reduced colistin use
Implementation of antimicrobial stewardship policies and colistin-use restrictions in multiple regions demonstrates feasibility of coordinated policy-driven interventions.	Limited laboratory capacity and lack of standardized susceptibility testing methods	Phase-out of colistin in agriculture, vaccination and biosecurity measures in livestock, and improved sanitation infrastructure	Global dissemination of high-risk clones and plasmids via travel, trade, and wildlife vectors
Increasing accessibility of sequencing and bioinformatics tools supports comprehensive resistome characterization and comparative genomics across sectors.	Data fragmentation between human, veterinary, and environmental sectors impede integrated responses	Network-informed targeting of hub taxa or plasmid backbones driving dissemination	Economic, regulatory, and ethical challenges may slow translation of microbiome-based, phage, or CRISPR-driven decolonization approaches into clinical or agricultural practice.
Growing recognition of One Health approaches in policy frameworks	Incomplete epidemiological data from LMICs creates geographic blind spots in global risk maps	Development of standardized trial protocols for horizontal gene transfer inhibition, microbiota restoration, and decolonization therapies could generate actionable clinical evidence.	Risk of delayed global coordination leading to entrenched resistance reservoirs

AMR—Antimicrobial resistance; CRISPR—Clustered regularly interspaced short palindromic repeats; LMICs—Low- and middle-income countries; *mcr*—Mobilized colistin resistance.

## References

[1] Thursby E., Juge N. (2017). Introduction to the human gut microbiota. Biochem. J..

[2] Sommer F., Bäckhed F. (2013). The gut microbiota—Masters of host development and physiology. Nat. Rev. Microbiol..

[3] Penders J., Stobberingh E.E., Savelkoul P.H.M., Wolffs P.F.G. (2013). The human microbiome as a reservoir of antimicrobial resistance. Front. Microbiol..

[4] Laxminarayan R., Duse A., Wattal C., Zaidi A.K.M., Wertheim H.F.L., Sumpradit N., Vlieghe E., Hara G.L., Gould I.M., Goossens H. (2013). Antibiotic resistance—The need for global solutions. Lancet Infect. Dis..

[5] Liu J.-H., Liu Y.-Y., Shen Y.-B., Yang J., Walsh T.R., Wang Y., Shen J. (2024). Plasmid-Mediated Colistin-Resistance Genes: *mcr*. Trends Microbiol..

[6] Poirel L., Jayol A., Nordmann P. (2017). Polymyxins: Antibacterial Activity, Susceptibility Testing, and Resistance Mechanisms Encoded by Plasmids or Chromosomes. Clin. Microbiol. Rev..

[7] De la Rosa-Carrillo D., Suárez-Cuartín G., Golpe R., Máiz Carro L., Martinez-Garcia M.A. (2022). Inhaled colistimethate sodium in the management of patients with bronchiectasis infected by *Pseudomonas aeruginosa*: A narrative review of current evidence. Infect. Drug Resist..

[8] Falagas M.E., Kasiakou S.K. (2005). Colistin: The revival of polymyxins for the management of multidrug-resistant Gram-negative bacterial infections. Clin. Infect. Dis..

[9] Liu Y.Y., Wang Y., Walsh T.R., Yi L.X., Zhang R., Spencer J., Doi Y., Tian G., Dong B., Huang X. (2016). Emergence of plasmid-mediated colistin resistance mechanism MCR-1 in animals and human beings in China: A microbiological and molecular biological study. Lancet Infect. Dis..

[10] Ling Z., Yin W., Shen Z., Wang Y., Shen J., Walsh T.R. (2020). Epidemiology of mobile colistin resistance genes mcr-1 to mcr-9. J. Antimicrob. Chemother..

[11] Wang R., van Dorp L., Shaw L.P., Bradley P., Wang Q., Wang X., Jin L., Zhang Q., Liu Y., Rieux A. (2018). The global distribution and spread of the mobilized colistin resistance gene *mcr*-1. Nat. Commun..

[12] National Center for Biotechnology Information (NCBI) Pathogen Detection Isolates Browser. mcr-11.1 AMR Genotype. https://www.ncbi.nlm.nih.gov/pathogens/isolates/#AMR_genotypes:mcr-11.1.

[13] Sun J., Zhang H., Liu Y.H., Feng Y. (2018). Towards understanding *mcr*-like colistin resistance. Trends Microbiol..

[14] Shen Y., Zhou H., Xu J., Wang Y., Zhang Q., Walsh T.R., Shao B., Wu C., Hu Y., Gao H. (2018). Anthropogenic and environmental factors associated with high incidence of *mcr*-1 carriage in humans across China. Nat. Microbiol..

[15] Partridge S.R., Kwong S.M., Firth N., Jensen S.O. (2018). Mobile genetic elements associated with antimicrobial resistance. Clin. Microbiol. Rev..

[16] Tawfick M.M., Elshamy A.A., Mohamed K.T., El Menofy N.G. (2022). Gut Commensal Escherichia coli, a High-Risk Reservoir of Transferable Plasmid-Mediated Antimicrobial Resistance Traits. Infect. Drug Resist..

[17] Smelikova E., Tkadlec J., Krutova M. (2022). How to: Screening for *mcr*-Mediated Resistance to Colistin. Clin. Microbiol. Infect..

[18] Turlej-Rogacka A., Xavier B.B., Janssens L., Lammens C., Zarkotou O., Pournaras S., Goossens H., Malhotra-Kumar S. (2018). Evaluation of Colistin Stability in Agar and Comparison of Four Methods for MIC Testing of Colistin. Eur. J. Clin. Microbiol. Infect. Dis..

[19] Witherell K.S., Price J., Bandaranayake A.D., Olson J., Call D.R. (2020). Circumventing Colistin Resistance by Combining Colistin and Antimicrobial Peptides to Kill Colistin-Resistant and Multidrug-Resistant Gram-Negative Bacteria. J. Glob. Antimicrob. Resist..

[20] Liu L., Feng Y., Zhang X., McNally A., Zong Z. (2017). New Variant of *mcr-3* in an Extensively Drug-Resistant *Escherichia coli* Clinical Isolate Carrying *mcr-1* and *blaNDM-5*. Antimicrob. Agents Chemother..

[21] Yin W., Ling Z., Dong Y., Qiao L., Shen Y., Liu Z., Wu Y., Li W., Zhang R., Walsh T.R. (2021). Mobile Colistin Resistance Enzyme MCR-3 Facilitates Bacterial Evasion of Host Phagocytosis. Adv. Sci..

[22] Carattoli A., Villa L., Feudi C., Curcio L., Orsini S., Luppi A., Pezzotti G., Magistrali C.F. (2017). Novel Plasmid-Mediated Colistin Resistance *mcr-4* Gene in *Salmonella* and *Escherichia coli*, Italy 2013, Spain and Belgium, 2015 to 2016. Eurosurveillance.

[23] Borowiak M., Fischer J., Hammerl J.A., Hendriksen R.S., Szabo I., Malorny B. (2017). Identification of a Novel Transposon-Associated Phosphoethanolamine Transferase Gene, *mcr-5*, Conferring Colistin Resistance in *d*-Tartrate Fermenting *Salmonella enterica* subsp. *enterica* Serovar Paratyphi B. J. Antimicrob. Chemother..

[24] AbuOun M., Stubberfield E.J., Duggett N.A., Kirchner M., Dormer L., Nunez-Garcia J., Randall L.P., Lemma F., Crook D.W., Teale C. (2017). *mcr-1* and *mcr-2 (mcr-6.1)* Variant Genes Identified in *Moraxella* Species Isolated from Pigs in Great Britain from 2014 to 2015. J. Antimicrob. Chemother..

[25] Phetburom N., Boueroy P., Chopjitt P., Hatrongjit R., Akeda Y., Hamada S., Nuanualsuwan S., Kerdsin A. (2021). *Klebsiella pneumoniae* Complex Harboring *mcr-1*, *mcr-7*, and *mcr-8* Isolates from Slaughtered Pigs in Thailand. Microorganisms.

[26] Eltai N.O., Kelly B., Al-Mana H.A., Ibrahim E.B., Yassine H.M., Al Thani A., Al Maslmani M., Lammens C., Xavier B.B., Malhotra-Kumar S. (2020). Identification of *mcr-8* in Clinical Isolates from Qatar and Evaluation of Their Antimicrobial Profiles. Front. Microbiol..

[27] Hatrongjit R., Wongsurawat T., Jenjaroenpun P., Chopjitt P., Boueroy P., Akeda Y., Okada K., Iida T., Hamada S., Kerdsin A. (2024). Genomic Analysis of Carbapenem- and Colistin-Resistant *Klebsiella pneumoniae* Complex Harbouring *mcr-8* and *mcr-9* from Individuals in Thailand. Sci. Rep..

[28] Carroll L.M., Gaballa A., Guldimann C., Sullivan G., Henderson L.O., Wiedmann M. (2019). Identification of Novel Mobilized Colistin Resistance Gene *mcr-9* in a Multidrug-Resistant, Colistin-Susceptible *Salmonella enterica* Serotype Typhimurium Isolate. mBio.

[29] Macesic N., Blakeway L.V., Stewart J.D., Hawkey J., Wyres K.L., Judd L.M., Wick R.R., Jenney A.W., Holt K.E., Peleg A.Y. (2021). Silent Spread of Mobile Colistin Resistance Gene *mcr-9.1* on IncHI2 ‘Superplasmids’ in Clinical Carbapenem-Resistant *Enterobacterales*. Clin. Microbiol. Infect..

[30] Wang C., Feng Y., Liu L., Wei L., Kang M., Zong Z. (2020). Identification of Novel Mobile Colistin Resistance Gene *mcr-10*. Emerg. Microbes Infect..

[31] Liao W., Cui Y., Quan J., Zhao D., Han X., Shi Q., Wang Q., Jiang Y., Du X., Li X. (2022). High Prevalence of Colistin Resistance and *mcr-9/10* Genes in *Enterobacter* spp. in a Tertiary Hospital over a Decade. Int. J. Antimicrob. Agents.

[32] Hernando-Amado S., Coque T.M., Baquero F., Martínez J.L. (2019). Defining and combating antibiotic resistance from One Health and Global Health perspectives. Nat. Microbiol..

[33] Campos-Madueno E.I., Moradi M., Eddoubaji Y., Shahi F., Moradi S., Bernasconi O.J., Moser A.I., Endimiani A. (2023). Intestinal colonization with multidrug-resistant *Enterobacterales*: Screening, epidemiology, clinical impact, and strategies to decolonize carriers. Eur. J. Clin. Microbiol. Infect. Dis..

[34] Gorrie C.L., Mirceta M., Wick R.R., Edwards D.J., Thomson N.R., Strugnell R.A., Pratt N., Garlick J.S., Watson K.M., Pilcher D.V. (2017). Gastrointestinal carriage is a major reservoir of *Klebsiella pneumoniae* infection in intensive care patients. Clin. Infect. Dis..

[35] Portal E.A.R., Sands K., Farley C., Boostrom I., Jones E., Barrell M., Carvalho M.J., Milton R., Iregbu K., Modibbo F. (2024). Characterisation of colistin resistance in Gram-negative microbiota of pregnant women and neonates in Nigeria. Nat. Commun..

[36] Rhouma M., Beaudry F., Thériault W., Letellier A. (2016). Colistin in pig production: Chemistry, mechanism of antibacterial action, microbial resistance emergence, and One Health perspectives. Front. Microbiol..

[37] EMA & ECDC Sales of Veterinary Antimicrobial Agents in 31 European Countries in 2018. EMA/24309/2020. https://share.google/5MgbK8Un3eCsnM8YH.

[38] Nobili G., La Bella G., Basanisi M.G., Damato A.M., Coppola R., Migliorelli R., Rondinone V., Leekitcharoenphon P., Bortolaia V., La Salandra G. (2022). Occurrence and Characterisation of Colistin-Resistant *Escherichia coli* in Raw Meat in Southern Italy in 2018–2020. Microorganisms.

[39] Ahmed S., Das T., Islam M.Z., Herrero-Fresno A., Biswas P.K., Olsen J.E. (2020). High prevalence of mcr-1-encoded colistin resistance in commensal *Escherichia coli* from broiler chicken in Bangladesh. Sci. Rep..

[40] Shi J., Zhu H., Liu C., Xie H., Li C., Cao X., Shen H. (2023). Epidemiological and genomic characteristics of global mcr-positive *Escherichia coli* isolates. Front. Microbiol..

[41] Azzam A., Salem H., Nazih M., Lotfy E.M., Hassan F.E., Khaled H. (2025). Prevalence, trends, and molecular insights into colistin resistance among gram-negative bacteria in Egypt: A systematic review and meta-analysis. Ann. Clin. Microbiol. Antimicrob..

[42] Anyanwu M.U., Jaja I.F., Nwobi O.C. (2020). Occurrence and characteristics of mobile colistin resistance (mcr) gene-containing isolates from the environment: A review. Int. J. Environ. Res. Public Health.

[43] Zhao J., Duan G., Chang J., Wang H., Zhu D., Li J., Zhu Y. (2025). Co-Exposure to Cyazofamid and Polymyxin E: Variations in Microbial Community and Antibiotic Resistance in the Soil–Animal–Plant System. Environ. Res..

[44] World Health Organization (WHO) (2015). Global Action Plan on Antimicrobial Resistance.

[45] Skov R.L., Monnet D.L. (2016). Plasmid-mediated colistin resistance (mcr-1 gene): Three months later, the story unfolds. Eurosurveillance.

[46] Andrade B.G.N., Goris T., Afli H., Coutinho F.H., Dávila A.M.R., Cuadrat R.R.C. (2021). Putative mobilized colistin resistance genes in the human gut microbiome. BMC Microbiol..

[47] Dalmasso G., Beyrouthy R., Brugiroux S., Ruppé E., Guillouard L., Bonnin V., Saint-Sardos P., Ghozlane A., Gaumet V., Barnich N. (2023). Genes mcr improve the intestinal fitness of pathogenic *Escherichia coli* and balance their lifestyle to commensalism. Microbiome.

[48] Avellán-Llaguno R.D., Xie A., Obeten A.U., Pan Z., Zhang Y., Ye G., Sun X., Huang Q. (2025). Composition, Antibiotic Resistance, and Functionality of the Gut Microbiome in Urban Cats. ACS Environ. Sci. Technol..

[49] Hassan J., Osman M., Xu T., Naas T., Schiff S.J., Mann D., Esseili M.A., Deng X., Kassem I.I. (2024). Monitoring Sewage and Effluent Water Is an Effective Approach for the Detection of the Mobile Colistin Resistance Genes (*mcr*) and Associated Bacterial Hosts in the Human Population and Environment in the USA. Environ. Pollut..

[50] Dwiyanto J., Huët M.A.L., Hussain M.H., Chong C.W., Rahman S., Choo S.W., Chin V.K., Rahman R.A., Chua E.G., Foo S.C. (2023). Social Demographics Determinants for Resistome and Microbiome Variation of a Multiethnic Community in Southern Malaysia. npj Biofilms Microbiomes.

[51] Li C., Chen J., Li S.C. (2020). Understanding Horizontal Gene Transfer Network in Human Gut Microbiota. Gut Pathog..

[52] Hendriksen R.S., Munk P., Njage P., van Bunnik B., McNally L., Lukjancenko O., Röder T., Nieuwenhuijse D., Pedersen S.K., Kjeldgaard J. (2019). Global Monitoring of Antimicrobial Resistance Based on Metagenomics Analyses of Urban Sewage. Nat. Commun..

[53] Zhang A.N., Gaston J.M., Dai C.L., Zhao S., Poyet M., Groussin M., Yin X., Li L.-G., van Loosdrecht M.C.M., Topp E. (2021). An Omics-Based Framework for Assessing the Health Risk of Antimicrobial Resistance Genes. Nat. Commun..

[54] Lee D.-H., Cha J.-H., Kim D.-W., Lee K., Kim Y.-S., Oh H.-Y., Cho Y.-H., Cha C.-J. (2022). Colistin-degrading proteases confer collective resistance to microbial communities during polymicrobial infections. Microbiome.

[55] Barlaam A., Parisi A., Spinelli E., Caruso M., Di Taranto P., Normanno G. (2019). Global Emergence of Colistin-Resistant *Escherichia coli* in Food Chains and Associated Food Safety Implications: A Review. J. Food Prot..

[56] Guo L., Zhang D., Fu S., Zhang J., Zhang X., He J., Peng C., Zhang Y., Qiu Y., Ye C. (2021). Metagenomic Sequencing Analysis of the Effects of Colistin Sulfate on the Pig Gut Microbiome. Front. Vet. Sci..

[57] Nation R.L., Li J., Cars O., Couet W., Dudley M.N., Kaye K.S., Mouton J.W., Paterson D.L., Tam V.H., Theuretzbacher U. (2015). Framework for optimisation of the clinical use of colistin and polymyxin B: The Prato polymyxin consensus. Lancet Infect. Dis..

[58] Li L., Wang Q., Gao Y., Liu L., Duan Y., Mao D., Luo Y. (2021). Colistin and amoxicillin combinatorial exposure alters the human intestinal microbiota and antibiotic resistome in the simulated human intestinal microbiota. Sci. Total Environ..

[59] Patangia D.V., Ryan C.A., Dempsey E., Ross R.P., Stanton C. (2022). Impact of antibiotics on the human microbiome and consequences for host health. MicrobiologyOpen.

[60] Lathakumari R.H., Vajravelu L.K., Satheesan A., Ravi S., Thulukanam J. (2024). Antibiotics and the gut microbiome: Understanding the impact on human health. Med. Microecol..

[61] Binsker U., Käsbohrer A., Hammerl J.A. (2021). Global colistin use: A review of the emergence of resistant *Enterobacterales* and the impact on their genetic basis. FEMS Microbiol. Rev..

[62] Shayista H., Prasad M.N.N., Raj S.N., Prasad A., Lakshmi S., Ranjini H.K., Manju K., Ravikumara, Chouhan R.S., Khohlova O.Y. (2025). Complexity of Antibiotic Resistance and Its Impact on Gut Microbiota Dynamics. Eng. Microbiol..

[63] Li R., Xie M., Zhang J., Yang Z., Liu L., Liu X., Zheng Z., Chan E.W., Chen S. (2017). Genetic Characterization of *mcr-1*-Bearing Plasmids to Depict Molecular Mechanisms Underlying Dissemination. J. Antimicrob. Chemother..

[64] Wang Y., Tian G.-B., Zhang R., Shen Y., Tyrrell J.M., Huang X., Zhou H., Lei L., Li H., Doi Y. (2017). Prevalence, Risk Factors, Outcomes, and Molecular Epidemiology of *mcr-1*-Positive Enterobacteriaceae in Patients and Healthy Adults from China: An Epidemiological and Clinical Study. Lancet Infect. Dis..

[65] Harmer C.J., Hall R.M. (2024). IS26 and the IS6 family: Versatile resistance gene movers and genome reorganizers. Microbiol. Mol. Biol. Rev..

[66] Yin W., Li H., Shen Y., Liu Z., Wang S., Shen Z., Zhang R., Walsh T.R., Shen J., Wang Y. (2017). Novel Plasmid-Mediated Colistin Resistance Gene *mcr-3* in *Escherichia coli*. mBio.

[67] Anyanwu M.U., Jaja I.F., Nwobi O.C., Mgbeahuruike A.C., Ikpendu C.N., Okafor N.A., Oguttu J.W. (2022). Epidemiology and Traits of Mobile Colistin Resistance (mcr) Gene-Bearing Organisms from Horses. Microorganisms.

[68] Beaber J.W., Hochhut B., Waldor M.K. (2004). SOS response promotes horizontal dissemination of antibiotic resistance genes. Nature.

[69] Kosterlitz O., Muñiz Tirado A., Wate C., Elg C., Bozic I., Top E.M., Kerr B. (2022). Estimating the Transfer Rates of Bacterial Plasmids with an Adapted Luria–Delbrück Fluctuation Analysis. PLoS Biol..

[70] Ridenhour B.J., Metzger G.A., France M., Gliniewicz K., Millstein J., Forney L.J., Top E.M. (2017). Persistence of Antibiotic Resistance Plasmids in Bacterial Biofilms. Evol. Appl..

[71] Stalder T., Press M.O., Sullivan S., Liachko I., Top E.M. (2019). Linking the Resistome and Plasmidome to the Microbiome. ISME J..

[72] Yaffe E., Relman D.A. (2020). Tracking Microbial Evolution in the Human Gut Using Hi-C Reveals Extensive Horizontal Gene Transfer, Persistence and Adaptation. Nat. Microbiol..

[73] Tamburini F.B., Andermann T.M., Bhatt A.S. (2016). The microbiome in early life: Implications for health outcomes. Nat. Med..

[74] Catassi G., Garcia Mateo S., Occhionero A.S., Esposito C., Giorgio V., Aloi M., Gasbarrini A., Cammarota G., Ianiro G. (2024). The importance of gut microbiome in the perinatal period. Eur. J. Pediatr..

[75] Li W., Tapiainen T., Brinkac L., Lorenzi H.A., Moncera K., Tejesvi M.V., Salo J., Nelson K.E. (2020). Vertical transmission of gut microbiome and antimicrobial resistance genes in infants exposed to antibiotics at birth. J. Infect. Dis..

[76] Rallis D., Giapros V., Serbis A., Baltogianni M. (2023). Fighting Antimicrobial Resistance in Neonatal Intensive Care Units: Rational Use of Antibiotics in Neonatal Sepsis. Antibiotics.

[77] Nakwan N., Chokephaibulkit K., Imberti R. (2019). The Use of Colistin for the Treatment of Multidrug-Resistant Gram-Negative Infections in Neonates and Infants: A Review of the Literature. Pediatr. Infect. Dis. J..

[78] Fouhy F., Watkins C., Hill C.J., O’Shea C.A., Nagle B., Dempsey E.M., O’Toole P.W., Stanton C., Ross R.P., Ryan C.A. (2019). Perinatal factors affect the gut microbiota up to four years after birth. Nat. Commun..

[79] Tanaka M., Nakayama J. (2017). Development of the gut microbiota in infancy and its impact on health in later life. Allergol. Int..

[80] Song K., Jin L., Cai M., Wang Q., Wu X., Wang S., Zhang Y., Li H., Chen Z., Liu P. (2024). Decoding the Origins, Spread, and Global Risks of *mcr-9* Gene. eBioMedicine.

[81] Farooq S., Talat A., Khan A.U. (2024). Letter to the Editor: Identification of *mcr-9.1* and *mcr-10.1* Colistin Resistance Genes in Neonates from Publicly Available Gut Metagenomic Data. Microb. Drug Resist..

[82] Lee Y.Q., Kamar A.A., Velayuthana R.D., Chong C.W., Teh C.S.J. (2021). Clonal relatedness in the acquisition of intestinal carriage and transmission of multidrug resistant (MDR) *Klebsiella pneumoniae* and *Escherichia coli* and its risk factors among preterm infants admitted to the neonatal intensive care unit (NICU). Pediatr. Neonatol..

[83] Bamford A., Masini T., Williams P., Sharland M., Gigante V., Dixit D., Sati H., Huttner B., Bin Nisar Y., Cappello B. (2024). Tackling the Threat of Antimicrobial Resistance in Neonates and Children: Outcomes from the First WHO-Convened Paediatric Drug Optimisation Exercise for Antibiotics. Lancet Child Adolesc. Health.

[84] Reyman M., van Houten M.A., Watson R.L.J., Chu M.L.J.N., Arp K., de Waal W.J., Schiering I., Plötz F.B., Willems R.J.L., van Schaik W. (2022). Effect of early-life antibiotics for suspected neonatal sepsis on gut microbiome trajectory and resistance gene profiles: A prospective randomized study. Nat. Commun..

[85] Shao Y., Forster S.C., Tsaliki E., Vervier K., Strang A., Simpson N., Kumar N., Stares M.D., Rodger A., Brocklehurst P. (2019). Stunted Microbiota and Opportunistic Pathogen Colonization in Caesarean-Section Birth. Nature.

[86] Kiu R., Darby E.M., Alcon-Giner C., Acuna-Gonzalez A., Camargo A., Lamberte L.E., Phillips S., Sim K., Shaw A.G., Clarke P. (2025). Impact of Early Life Antibiotic and Probiotic Treatment on Gut Microbiome and Resistome of Very-Low-Birth-Weight Preterm Infants. Nat. Commun..

[87] Korpela K., Helve O., Kolho K.L., Saisto T., Skogberg K., Dikareva E., Stefanovic V., Salonen A., Andersson S., de Vos W.M. (2020). Maternal fecal microbiota transplantation in cesarean-born infants rapidly restores normal gut microbial development: A proof-of-concept study. Cell.

[88] Dutra S.V.O., Sarkar A., Yoo J.Y., Shaffer-Hudkins E., Groer M. (2024). Premature Infant Gut Microbiome Relationships with Childhood Behavioral Scales: Preliminary Insights. Front. Nutr..

[89] Sarkar A., Yoo J.Y., Dutra S.V.O., Morgan K.H., Groer M. (2021). The Association between Early-Life Gut Microbiota and Long-Term Health and Diseases. J. Clin. Med..

[90] Olaitan A.O., Morand S., Rolain J.M. (2014). Mechanisms of polymyxin resistance: Acquired and intrinsic resistance in bacteria. Front. Microbiol..

[91] De Angelis G., Del Giacomo P., Posteraro B., Sanguinetti M., Tumbarello M. (2020). Molecular mechanisms, epidemiology, and clinical importance of β-lactam resistance in *Enterobacteriaceae*. Int. J. Mol. Sci..

[92] Tamma P.D., Aitken S.L., Bonomo R.A., Mathers A.J., van Duin D., Clancy C.J. (2021). Infectious Diseases Society of America Guidance on the Treatment of AmpC β-Lactamase–Producing *Enterobacterales*. Clin. Infect. Dis..

[93] Caselli E., D’Accolti M., Soffritti I., Piffanelli M., Mazzacane S. (2018). Spread of *mcr-1*-Driven Colistin Resistance on Hospital Surfaces, Italy. Emerg. Infect. Dis..

[94] Nicolas-Chanoine M.H., Bertrand X., Madec J.Y. (2014). *Escherichia coli* ST131, an intriguing clonal group. Clin. Microbiol. Rev..

[95] D’Souza A.W., Boolchandani M., Patel S., Owens S.M., Siegel M., Mathur S., Engevik K., Atwood C., Rood J.I., Dantas G. (2021). Destination shapes antibiotic resistance gene acquisitions, abundance increases, and diversity changes in Dutch travelers. Genome Med..

[96] Giani T., Sennati S., Antonelli A., Di Pilato V., di Maggio T., Mantella A., Niccolai C., Spinicci M., Monasterio J., Castellanos P. (2018). High Prevalence of Carriage of *mcr-1*-Positive Enteric Bacteria among Healthy Children from Rural Communities in the Chaco Region, Bolivia, September to October 2016. Eurosurveillance.

[97] Bich V.T.N., Thanh L.V., Thai P.D., Hoa N.T., Tuan P.Q., Hien V.T., Lan N.T., Anh P.H., Hanh T.T., Dung T.T. (2019). An exploration of the gut and environmental resistome in a community in northern Vietnam in relation to antibiotic use. Antimicrob. Resist. Infect. Control.

[98] Manzoor H., Hao L., Kayani M.U.R. (2025). Identification of Antibiotic Resistance Genes from Whole Genome and Metagenome Sequencing Datasets. One Health Adv..

[99] Salleh M.Z. (2025). Addressing antimicrobial resistance: Structural insights into cefiderocol’s mode of action and emerging resistance mechanisms. J. Infect. Public Health.

[100] Zhanel G.G., Cheung D., Adam H., Zelenitsky S., Golden A., Schweizer F., Gorityala B., Lagacé-Wiens P.R.S., Walkty A., Gin A.S. (2016). Review of eravacycline, a novel fluorocycline antibacterial agent. Drugs.

[101] Antonello R.M., Principe L., Maraolo A.E., Viaggi V., Pol R., Fabbiani M., Montagnani F., Lovecchio A., Luzzati R., Di Bella S. (2020). Fosfomycin as Partner Drug for Systemic Infection Management. A Systematic Review of Its Synergistic Properties from In Vitro and In Vivo Studies. Antibiotics.

[102] Karakonstantis S., Rousaki M., Kritsotakis E.I. (2022). Cefiderocol: Systematic Review of Mechanisms of Resistance, Heteroresistance and In Vivo Emergence of Resistance. Antibiotics.

[103] Klein S., Boutin S., Kocer K., Fiedler M.O., Störzinger D., Weigand M.A., Tan B., Richter D., Rupp C., Mieth M. (2022). Rapid Development of Cefiderocol Resistance in Carbapenem-Resistant *Enterobacter cloacae* during Therapy Is Associated with *cirA* Mutations. Clin. Infect. Dis..

[104] Wunderink R.G., Matsunaga Y., Ariyasu M., Clevenbergh P., Echols R., Kaye K.S., Kollef M., Menon A., Pogue J.M., Shorr A.F. (2020). Cefiderocol versus High-Dose, Extended-Infusion Meropenem for the Treatment of Gram-Negative Nosocomial Pneumonia (APEKS-NP): A Randomised, Double-Blind, Phase 3, Non-Inferiority Trial. Lancet Infect. Dis..

[105] Takemura M., Yamano Y., Matsunaga Y., Ariyasu M., Echols R., Nagata T.D. (2020). Characterization of Shifts in Minimum Inhibitory Concentrations during Treatment with Cefiderocol or Comparators in the Phase 3 CREDIBLE-CR and APEKS-NP Studies. Open Forum Infect. Dis..

[106] Mookherjee N., Anderson M.A., Haagsman H.P., Davidson D.J. (2020). Antimicrobial host defence peptides: Functions and clinical potential. Nat. Rev. Drug Discov..

[107] Lima P.G., Oliveira J.T.A., Amaral J.L., Freitas C.D.T., Souza P.F.N. (2021). Synthetic antimicrobial peptides: Characteristics, design, and potential as alternative molecules to overcome microbial resistance. Life Sci..

[108] Rieg S., Küpper M.F., de With K., Schwab F., Pfeifer Y., Heudorf U., Kola A., Seifert H., Klupp E.M., Bohnert J.A. (2015). Intestinal decolonization of *Enterobacteriaceae* producing extended-spectrum β-lactamases (ESBL): A retrospective observational study in patients at risk for infection and a brief review of the literature. BMC Infect. Dis..

[109] Xenofontos E., Renieris G., Kalogridi M., Papageorgiou E., Christodoulou S., Spanakis N., Rapti V., Triantafyllou I., Tsagris V., Giamarellou H. (2022). An animal model of limitation of gut colonization by carbapenemase-producing *Klebsiella pneumoniae* using rifaximin. Sci. Rep..

[110] Alagna L., Palomba E., Mangioni D., Bozzi G., Lombardi A., Ungaro R., Castelli V., Prati D., Vecchi M., Muscatello A. (2020). Multidrug-Resistant Gram-Negative Bacteria Decolonization in Immunocompromised Patients: A Focus on Fecal Microbiota Transplantation. Int. J. Mol. Sci..

[111] Shin J., Lee J.-H., Park S.-H., Cha B., Kwon K.S., Kim H., Shin Y.W. (2022). Efficacy and Safety of Fecal Microbiota Transplantation for Clearance of Multidrug-Resistant Organisms under Multiple Comorbidities: A Prospective Comparative Trial. Biomedicines.

[112] Nooij S., Vendrik K.E.W., Zwittink R.D., Ducarmon Q.R., Keller J.J., Kuijper E.J., Terveer E.M., Netherlands Donor Feces Bank Study Group (2024). Long-Term Beneficial Effect of Faecal Microbiota Transplantation on Colonisation of Multidrug-Resistant Bacteria and Resistome Abundance in Patients with Recurrent *Clostridioides difficile* Infection. Genome Med..

[113] Rasmussen T.S., Mao X., Forster S., Larsen S.B., Von Münchow A., Tranæs K.D., Brunse A., Larsen F., Castro Mejia J.L., Adamberg S. (2024). Overcoming Donor Variability and Risks Associated with Fecal Microbiota Transplants through Bacteriophage-Mediated Treatments. Microbiome.

[114] Bénard M.V., de Bruijn C.M.A., Fenneman A.C., Wortelboer K., Zeevenhoven J., Rethans B., Herrema H.J., van Gool T., Nieuwdorp M., Benninga M.A. (2022). Challenges and Costs of Donor Screening for Fecal Microbiota Transplantations. PLoS ONE.

[115] Mousavi S.M., Babakhani S., Moradi L., Karami S., Shahbandeh M., Mirshekar M., Mohebi S., Moghadam M.T. (2021). Bacteriophage as a Novel Therapeutic Weapon for Killing Colistin-Resistant MDR and XDR Gram-Negative Bacteria: A Review. Curr. Microbiol..

[116] Geng X., Zhang Z.-D., Li Y.-X., Hao R.-C., Yang Y.-J., Liu X.-W., Li J.-Y. (2024). Fingolimod synergizes and reverses *Klebsiella pneumoniae* resistance to colistin. Front. Microbiol..

[117] Wang X., Loh B., Gordillo Altamirano F., Yu Y., Hua X., Leptihn S. (2021). Colistin-phage combinations decrease antibiotic resistance in *Acinetobacter baumannii* via changes in envelope architecture. Emerg. Microbes Infect..

[118] Malik D.J., Sokolov I.J., Vinner G.K., Mancuso F., Cinquerrui S., Vladisavljevic G.T., Clokie M.R.J., Garton N.J., Stapley A.G.F., Kirpichnikova A. (2017). Formulation, stabilisation and encapsulation of bacteriophage for phage therapy. Adv. Colloid Interface Sci..

[119] Nale J.Y., Spencer J., Hargreaves K.R., Buckley A.M., Trzepinski P., Douce G.R., Clokie M.R.J. (2016). Bacteriophage Combinations Significantly Reduce Clostridium difficile Growth In Vitro and Proliferation In Vivo. Antimicrob. Agents Chemother..

[120] Zhao M., Li H., Gan D., Wang M., Deng H., Yang Q.E. (2024). Antibacterial effect of phage cocktails and phage-antibiotic synergy against pathogenic *Klebsiella pneumoniae*. mSystems.

[121] Khambhati K., Bhattacharjee G., Gohil N., Dhanoa G.K., Sagona A.P., Mani I., Bui N.L., Chu D.-T., Karapurkar J.K., Jang S.H. (2022). Phage engineering and phage-assisted CRISPR-Cas delivery to combat multidrug-resistant pathogens. Bioeng. Transl. Med..

[122] Kim M.K., Suh G.A., Cullen G.D., Perez Rodriguez S., Dharmaraj T., Chang T.H.W., Li Z., Chen Q., Green S.I., Lavigne R. (2025). Bacteriophage Therapy for Multidrug-Resistant Infections: Current Technologies and Therapeutic Approaches. J. Clin. Investig..

[123] Kou X., Yang Y., Zheng R. (2024). Challenges and Opportunities of Phage Therapy for *Klebsiella pneumoniae* Infections. Appl. Environ. Microbiol..

[124] Van Nieuwenhuyse B., Merabishvili M., Goeders N., Vanneste K., Bogaerts B., de Jode M., Ravau J., Wagemans J., Belkhir L., Van der Linden D. (2024). Phage-Mediated Digestive Decolonization in a Gut-On-A-Chip Model: A Tale of Gut-Specific Bacterial Prosperity. Viruses.

[125] Ahmed M.M., Kayode H.H., Okesanya O.J., Ukoaka B.M., Eshun G., Mourid M.R., Adigun O.A., Ogaya J.B., Mohamed Z.O., Lucero-Prisno III D.E. (2024). CRISPR-Cas systems in the fight against antimicrobial resistance: Current status, potentials, and future directions. Infect. Drug Resist..

[126] Kadkhoda H., Gholizadeh P., Samadi Kafil H., Ghotaslou R., Pirzadeh T., Ahangarzadeh Rezaee M., Nabizadeh E., Feizi H., Aghazadeh M. (2024). Role of CRISPR-Cas systems and anti-CRISPR proteins in bacterial antibiotic resistance. Heliyon.

[127] Neil K., Allard N., Roy P., Grenier F., Menendez A., Burrus V., Rodrigue S. (2021). High-Efficiency Delivery of CRISPR-Cas9 by Engineered Probiotics Enables Precise Microbiome Editing. Mol. Syst. Biol..

[128] Duan C., Cao J., Gu J., Zhang Y., Wang Q., Liang J., Xu L., Zhang T. (2021). Harnessing the CRISPR-Cas Systems to Combat Antimicrobial Resistance. Front. Microbiol..

[129] Lam K.N., Spanogiannopoulos P., Soto-Perez P., Alexander M., Nalley M.J., Bisanz J.E., Nayak R.R., Weakley A.M., Yu F.B., Turnbaugh P.J. (2021). Phage-Delivered CRISPR-Cas9 for Strain-Specific Depletion and Genomic Deletions in the Gut Microbiome. Cell Rep..

[130] Pursey E., Sünderhauf D., Gaze W.H., Westra E.R., van Houte S. (2018). CRISPR-Cas Antimicrobials: Challenges and Future Prospects. PLoS Pathog..

[131] de la Fuente Tagarro C., Martín-González D., de Lucas A., Bordel S., Santos-Beneit F. (2024). Current Knowledge on CRISPR Strategies against Antimicrobial-Resistant Bacteria. Antibiotics.

[132] Sachdeva A., Tomar T., Malik T., Bains A., Karnwal A. (2025). Exploring probiotics as a sustainable alternative to antimicrobial growth promoters: Mechanisms and benefits in animal health. Front. Sustain. Food Syst..

[133] Paranga T.G., Mitu I., Pavel-Tanasa M., Rosu M.F., Miftode I.-L., Constantinescu D., Obreja M., Plesca C.E., Miftode E. (2024). Cytokine Storm in COVID-19: Exploring IL-6 Signaling and Cytokine-Microbiome Interactions as Emerging Therapeutic Approaches. Int. J. Mol. Sci..

[134] Anton-Păduraru D.-T., Trofin F., Nastase E.V., Miftode R.S., Miftode I.-L., Trandafirescu M.F., Cojocaru E., Țarcă E., Mindru D.E., Dorneanu O.S. (2024). The Role of the Gut Microbiota in Anorexia Nervosa in Children and Adults—Systematic Review. Int. J. Mol. Sci..

[135] Zollner-Schwetz I., Scarpatetti M., Pichler G., Pux C., Klymiuk I., Trajanoski S., Krause R. (2020). Effect of a multispecies probiotic on intestinal and skin colonization by multidrug-resistant gram-negative bacteria in patients in a long-term care facility: A pilot study. Nutrients.

[136] Karbalaei M., Keikha M. (2022). Probiotics and intestinal decolonization of antibiotic-resistant microorganisms; a reality or fantasy?. Ann. Med. Surg..

[137] Everard A., Belzer C., Geurts L., Ouwerkerk J.P., Druart C., Bindels L.B., Guiot Y., Derrien M., Muccioli G.G., Delzenne N.M. (2013). Cross-Talk Between *Akkermansia muciniphila* and the Intestinal Epithelium Controls Diet-Induced Obesity. Proc. Natl. Acad. Sci. USA.

[138] Chelakkot C., Choi Y., Kim D.-K., Park H.T., Ghim J., Kwon Y., Jeon J., Kim M.-S., Jee Y.-K., Gho Y.S. (2018). *Akkermansia muciniphila*-derived extracellular vesicles influence gut permeability through the regulation of tight junctions. Exp. Mol. Med..

[139] Zheng M., Han R., Yuan Y., Xing Y., Zhang W., Sun Z., Liu Y., Li J., Mao T. (2023). The role of *Akkermansia muciniphila* in inflammatory bowel disease: Current knowledge and perspectives. Front. Immunol..

[140] Ljungquist O., Kampmann C., Resman F., Riesbeck K., Tham J. (2020). Probiotics for Intestinal Decolonization of ESBL-Producing Enterobacteriaceae: A Randomized, Placebo-Controlled Clinical Trial. Clin. Microbiol. Infect..

[141] Rauseo A.M., Hink T., Reske K.A., Seiler S.M., Bommarito K.M., Fraser V.J., Burnham C.A.D., Dubberke E.R. (2022). A Randomized Controlled Trial of *Lactobacillus rhamnosus* GG on Antimicrobial-Resistant Organism Colonization. Infect. Control Hosp. Epidemiol..

[142] Hung Y.-P., Lee C.-C., Lee J.-C., Tsai P.-J., Hsueh P.-R., Ko W.-C. (2021). The Potential of Probiotics to Eradicate Gut Carriage of Pathogenic or Antimicrobial-Resistant Enterobacterales. Antibiotics.

[143] Newman A.M., Arshad M. (2020). The Role of Probiotics, Prebiotics and Synbiotics in Combating Multidrug-Resistant Organisms. Clin. Ther..

[144] Depommier C., Everard A., Druart C., Plovier H., Van Hul M., Vieira-Silva S., Falony G., Raes J., Maiter D., Delzenne N.M. (2019). Supplementation with *Akkermansia muciniphila* in Overweight and Obese Human Volunteers: A Proof-of-Concept Exploratory Study. Nat. Med..

[145] Barberio D., Microbiome Therapeutics Innovation Group (2024). Navigating Regulatory and Analytical Challenges in Live Biotherapeutic Product Development and Manufacturing. Front. Microbiomes.

[146] Denkel L.A., Gastmeier P., Reichardt C., Köck R., Vehreschild M., Seifert H., de With K., Kern W.V., Weichert S., Knobloch J.K. (2024). Can probiotics trigger a paradigm shift for cleaning healthcare?. Antimicrob. Resist. Infect. Control..

[147] National Institutes of Health Enhancing Mechanistic Research on Precision Probiotics (PAR-25-211). NIH Grants Funding 2024. https://grants.nih.gov/grants/guide/pa-files/PAR-25-211.html.

[148] del Olmo M., Andreu C. (2025). Current Status of the Application of Antimicrobial Peptides and Their Conjugated Derivatives. Molecules.

[149] Xu T., Fang D., Li F., Wang Z., Liu Y. (2025). Vitamin B6 Resensitizes *mcr*-Carrying Gram-Negative Bacteria to Colistin. Commun. Biol..

[150] Mahlapuu M., Håkansson J., Ringstad L., Björn C. (2016). Antimicrobial Peptides: An Emerging Category of Therapeutic Agents. Front. Cell. Infect. Microbiol..

[151] Magana M., Pushpanathan M., Santos A.L., Leanse L., Fernandez M., Ioannidis A., Giulianotti M.A., Apidianakis Y., Bradfute S., Ferguson A.L. (2020). The Value of Antimicrobial Peptides in the Age of Resistance. Lancet Infect. Dis..

[152] Pelgrift R.Y., Friedman A.J. (2013). Nanotechnology as a Therapeutic Tool to Combat Microbial Resistance. Adv. Drug Deliv. Rev..

[153] Arnold A., McLellan S., Stokes J.M. (2025). How AI Can Help Us Beat AMR. npj Antimicrob. Resist..

[154] Li Y., Cui X., Yang X., Liu G., Zhang J. (2024). Artificial Intelligence in Predicting Pathogenic Microorganisms’ Antimicrobial Resistance: Challenges, Progress, and Prospects. Front. Cell. Infect. Microbiol..

[155] Valavarasu S., Sangu Y., Mahapatra T. (2025). Prediction of Antibiotic Resistance from Antibiotic Susceptibility Testing Results from Surveillance Data Using Machine Learning. Sci. Rep..

[156] Lin B., Yan S., Zhen B. (2025). A Machine Learning Method for Predicting Molecular Antimicrobial Activity. Sci. Rep..

[157] Renz J., Dauda K.A., Aga O.N.L., Diaz-Uriarte R., Löhr I.H., Blomberg B., Johnston I.G. (2024). Evolutionary Accumulation Modelling in AMR: Machine Learning to Infer and Predict Evolutionary Dynamics of Multi-Drug Resistance. arXiv.

[158] López-Cortés X.A., Manríquez-Troncoso J.M., Hernández-García R., Peralta D. (2024). MSDeepAMR: Antimicrobial Resistance Prediction Based on Deep Neural Networks and Transfer Learning. Front. Microbiol..

[159] Zhou G., Janarthanan S., Lu Y., Hu P. (2025). CL-MFAP: A Contrastive Learning-Based Multimodal Foundation Model for Molecular Property Prediction and Antibiotic Screening. arXiv.

[160] Theodosiou A.A., Read R.C. (2023). Artificial intelligence, machine learning and deep learning: Potential resources for the infection clinician. J. Infect..

[161] Kim J.I., Maguire F., Tsang K.K., Gouliouris T., Peacock S.J., McAllister T.A., McArthur A.G., Beiko R.G. (2022). Machine Learning for Antimicrobial Resistance Prediction: Current Practice, Limitations, and Clinical Perspective. Clin. Microbiol. Rev..

[162] Shields R.K., Nguyen M.H., Press E.G., Chen L., Kreiswirth B.N., Clancy C.J. (2017). In vitro selection of meropenem resistance among ceftazidime-avibactam-resistant, meropenem-susceptible *Klebsiella pneumoniae* isolates with variant KPC-3 carbapenemases. Antimicrob. Agents Chemother..

[163] Cannatelli A., Giani T., D’Andrea M.M., Di Pilato V., Arena F., Conte V., Tryfinopoulou K., Vatopoulos A., Rossolini G.M. (2014). MgrB inactivation is a common mechanism of colistin resistance in KPC-producing *Klebsiella pneumoniae*. Antimicrob. Agents Chemother..

[164] Sargianou M., Stathopoulos P., Vrysis C., Tzvetanova I.D., Falagas M.E. (2025). New β-Lactam/β-Lactamase Inhibitor Combination Antibiotics. Pathogens.

[165] El-Sokkary R., Erdem H., Kullar R., Pekok A.U., Amer F., Grgić S., Carevic B., El-Kholy A., Liskova A., Özdemir M. (2022). Self-Reported Antibiotic Stewardship and Infection Control Measures from 57 Intensive Care Units: An International ID-IRI Survey. J. Infect. Public Health.

[166] Kempf I., Jouy E., Chauvin C. (2016). Colistin use and colistin resistance in bacteria from animals. Int. J. Antimicrob. Agents.

[167] Aidara-Kane A., Andremont A., Collignon P., Angulo F.J., Conly J.M., Minato Y., Silbergeld E.K., McEwen S.A., Balkhy H., Friedman C. (2018). World Health Organization guidelines on use of medically important antimicrobials in food-producing animals. Antimicrob. Resist. Infect. Control..

[168] World Health Organization (2022). Global Antimicrobial Resistance and Use Surveillance System (GLASS) Report.

[169] Do P.C., Assefa Y.A., Batikawai S.M., Reid S.A. (2023). Strengthening Antimicrobial Resistance Surveillance Systems: A Scoping Review. BMC Infect. Dis..

[170] Nguyen T.N., Khong D.T., Le H.V., Tran H.T., Phan Q.N., Le H.T.T., Kawahara R., Yamamoto Y. (2021). Quantitative analysis of colistin-resistant *Escherichia coli* in retail meat from local Vietnamese markets. Biomed. Res. Int..

[171] Talat A., Usmani A., Khan A.U. (2022). Detection of *E. coli* IncX1 Plasmid-Mediated mcr-5.1 Gene in an Indian Hospital Sewage Water Using Shotgun Metagenomic Sequencing: A First Report. Microbiol. Drug Resist..

[172] Trung N.V., Matamoros S., Carrique-Mas J.J., Nghia N.H., Nhung N.T., Chieu T.T.B., Mai H.H., van Rooijen W., Campbell J., Wagenaar J.A. (2017). Zoonotic transmission of mcr-1 colistin resistance gene from small-scale poultry farms, Vietnam. Emerg. Infect. Dis..

[173] Schmitt H., Blaak H., Kemper M., de Roda Husman A.M. (2023). Wastewater Based Surveillance of AMR in the Netherlands.

[174] Miftode I.-L., Leca D., Miftode R.-S., Roşu F., Plesca C., Loghin I., Timpau A.S., Mitu I., Mititiuc I., Dorneanu O. (2023). The Clash of the Titans: COVID-19, Carbapenem-Resistant Enterobacterales, and First mcr-1-Mediated Colistin Resistance in Humans in Romania. Antibiotics.

[175] Maciuca I.E., Cummins M.L., Cozma A.P., Rimbu C.M., Guguianu E., Panzaru C., Licker M., Szekely E., Flonta M., Djordjevic S.P. (2019). Genetic Features of mcr-1-Mediated Colistin Resistance in CMY-2-Producing *Escherichia coli* from Romanian Poultry. Front. Microbiol..

[176] Schaumburg F., Sertic S.M., Correa-Martinez C., Mellmann A., Köck R., Becker K. (2019). Acquisition and colonization dynamics of antimicrobial-resistant bacteria during international travel: A prospective cohort study. Clin. Microbiol. Infect..

[177] Mistry K., Thumbi D., Li X.R., Charlebois A., Avery B.P., Deckert A.E., Cormier A.C., Murphy C., Kearney A., Campbell J. (2025). One Health Study of Mobile Colistin Resistance (*mcr*) in *Salmonella enterica* in Canada, 2017–2022. Microbiol. Spectr..

[178] Wang P., Smith A.L. (2023). Emergence of mobile colistin resistance genes within Los Angeles County wastewater. Environ. Sci. Technol. Lett..

[179] De La Cadena E., Mahecha M., Velandia A.M., García-Betancur J.C., Rojas L.J., Porras J., Pallares C., Villegas M.V. (2023). Identification of mcr-1 Genes and Characterization of Resistance Mechanisms to Colistin in *Escherichia coli* Isolates from Colombian Hospitals. Antibiotics.

[180] Lentz S.A.M., Dalmolin T.V., Barth A.L., Martins A.F. (2021). mcr-1 gene in Latin America: How is it disseminated among humans, animals, and the environment?. Front. Public Health.

[181] Bastidas-Caldes C., de Waard J.H., Salgado M.S., Villacís M.J., Coral-Almeida M., Yamamoto Y., Calvopiña M. (2022). Worldwide Prevalence of mcr-mediated Colistin-Resistance *Escherichia coli* in Isolates of Clinical Samples, Healthy Humans, and Livestock—A Systematic Review and Meta-Analysis. Pathogens.

[182] Tacconelli E., Carrara E., Savoldi A., Harbarth S., Mendelson M., Monnet D.L., Pulcini C., Kahlmeter G., Kluytmans J., Carmeli Y. (2018). Discovery, research, and development of new antibiotics: The WHO priority list. Lancet Infect. Dis..

[183] Otter J.A., Mutters N.T., Tacconelli E., Gikas A., Holmes A.H. (2015). Controversies in guidelines for the control of multidrug-resistant Gram-negative bacteria in EU hospitals. Clin. Microbiol. Infect..

[184] Sanabria A.M., Janice J., Hjerde E., Simonsen G.S., Hanssen A.-M. (2021). Shotgun-Metagenomics Based Prediction of Antibiotic Resistance and Virulence Determinants in *Staphylococcus aureus* from Periprosthetic Tissue on Blood Culture Bottles. Sci. Rep..

[185] Chen X., Yin X., Xu X., Zhang T. (2025). Species-Resolved Profiling of Antibiotic Resistance Genes in Complex Metagenomes through Long-Read Overlapping with Argo. Nat. Commun..

[186] Rajput V., Pramanik R., Nannaware K., Shah P., Bhalerao A., Jain N., Shashidhara L.S., Kamble S., Dastager S., Dharne M. (2025). Metagenomics-Based Longitudinal Monitoring of Antibiotic Resistome and Microbiome in the Inlets of Wastewater Treatment Plants in an Indian Megacity. Sci. Total Environ..

[187] Naylor N.R., Hasso-Agopsowicz M., Kim C., Ma Y., Frost I., Abbas K., Aguilar G., Fuller N., Robotham J.V., Jit M. (2025). The Global Economic Burden of Antibiotic-Resistant Infections and the Potential Impact of Bacterial Vaccines: A Modelling Study. BMJ Glob. Health.

[188] Dadashi M., Sameni F., Bostanshirin N., Yaslianifard S., Khosravi-Dehaghi N., Nasiri M.J., Goudarzi M., Hashemi A., Hajikhani B. (2022). Global prevalence and molecular epidemiology of *mcr*-mediated colistin resistance in *Escherichia coli* clinical isolates: A systematic review. J. Glob. Antimicrob. Resist..

